# Controlling Malaria Using Livestock-Based Interventions: A One Health Approach

**DOI:** 10.1371/journal.pone.0101699

**Published:** 2014-07-22

**Authors:** Ana O. Franco, M. Gabriela M. Gomes, Mark Rowland, Paul G. Coleman, Clive R. Davies

**Affiliations:** 1 Instituto Gulbenkian de Ciência, Oeiras, Portugal; 2 Faculty of Infectious and Tropical Diseases, London School of Hygiene and Tropical Medicine, London, United Kingdom; Swiss Tropical & Public Health Institute, Switzerland

## Abstract

Where malaria is transmitted by zoophilic vectors, two types of malaria control strategies have been proposed based on animals: using livestock to divert vector biting from people (zooprophylaxis) or as baits to attract vectors to insecticide sources (insecticide-treated livestock). Opposing findings have been obtained on malaria zooprophylaxis, and despite the success of an insecticide-treated livestock trial in Pakistan, where malaria vectors are highly zoophilic, its effectiveness is yet to be formally tested in Africa where vectors are more anthropophilic. This study aims to clarify the different effects of livestock on malaria and to understand under what circumstances livestock-based interventions could play a role in malaria control programmes. This was explored by developing a mathematical model and combining it with data from Pakistan and Ethiopia. Consistent with previous work, a zooprophylactic effect of untreated livestock is predicted in two situations: if vector population density does not increase with livestock introduction, or if livestock numbers and availability to vectors are sufficiently high such that the increase in vector density is counteracted by the diversion of bites from humans to animals. Although, as expected, insecticide-treatment of livestock is predicted to be more beneficial in settings with highly zoophilic vectors, like South Asia, we find that the intervention could also considerably decrease malaria transmission in regions with more anthropophilic vectors, like *Anopheles arabiensis* in Africa, under specific circumstances: high treatment coverage of the livestock population, using a product with stronger or longer lasting insecticidal effect than in the Pakistan trial, and with small (ideally null) repellency effect, or if increasing the attractiveness of treated livestock to malaria vectors. The results suggest these are the most appropriate conditions for field testing insecticide-treated livestock in an Africa region with moderately zoophilic vectors, where this intervention could contribute to the integrated control of malaria and livestock diseases.

## Introduction

In the last few decades there has been increasing recognition of the need for an integrated public health and veterinary approach, accounting for the surrounding social-ecological system, to face many of the most challenging disease threats: the so-called ‘One Health’ approach [1]. Broadly speaking, animals play an important role in the epidemiology of several of the most important diseases of man, where they can act as a reservoir source for infectious pathogens, and/or a source of blood-meal to arthropod vectors of human disease. The recognition of this relationship has led to the implementation of human disease control strategies targeted at animal populations. These control opportunities have been investigated both empirically and theoretically. Yet, our knowledge on what determines the public health benefits of many of these veterinary interventions remains limited.

A case study of the ‘One Health’ concept is human malaria in regions where its mosquito vectors (*Anopheles* spp.) also feed on animals, since the presence of livestock close to the household can affect the rate of vector-human contacts and consequently the risk of disease transmission among people. As the *Plasmodium* malaria parasites that infect humans are not infective to livestock, it has since long been proposed that animals could be used to divert the malaria vector biting from humans, a control intervention known as zooprophylaxis [2], [3]. However, despite the large number of studies performed worldwide for over a century to try to assess the value of this strategy in the fight against malaria (reviewed in [4], [5], [6], [7], [8], [9]), the available evidence is still contradictory and no consensus exists on the prophylactic effect of animals. Indeed, although in several situations the presence of livestock has been referred to as a protective factor for malaria vector-human contact and/or disease, such as in Papua New Guinea [10], [11] and Sri Lanka [12], the opposite has been reported in various other studies, where livestock were shown to be a risk factor, such as Pakistan [13], [14], Philippines [15], [16], and Ethiopia [17], [18] (throughout this work the term livestock is used to refer to cattle and other domestic large and small ruminants - buffalos, sheep, goats -, as well as donkeys, horses, and swine).

The apparently contradictory outcomes of the numerous studies conducted result from a combination of several possible effects of livestock on malaria. On one hand, livestock may divert the blood-seeking mosquito vectors from humans, thereby decreasing the biting on people [10], [11], [19] and, as a result, decreasing the transmission of the malaria parasite [20] and preventing its amplification in people (i.e. the basis for the zooprophylaxis concept). But on the other hand, livestock can provide additional blood-sources and/or larval breeding sites [21], [22], [23], [24], [25], which can increase vector survival and/or density [26], consequently increasing the probability of the vector surviving the parasite extrinsic incubation period and becoming infectious, as well as increasing biting on people [2], [6], [27]. Additionally, livestock may attract more mosquitoes, which, once in the vicinity of the human dwellings, may end up biting humans rather than animals [14], [15], [16], [18]. The resulting net impact of livestock on malaria risk therefore depends on the relative contribution of each of those effects.

In areas where the presence of livestock near people increases malaria transmission, an apparently simple solution could be to change livestock management in order to deploy the animals away from people's houses, between village and vector breeding site [7]. However, in Pakistan as well as in some Ethiopian regions, for instance, this is not likely to be a feasible strategy, given that livestock are such an important source of household income that people prefer to keep the animals near their houses to prevent them from being stolen [13], [28], [29], [30] and to facilitate husbandry practices, such as milking the lactating animals. An alternative solution has therefore been proposed: target the non-human host of the zoophilic mosquito, by treating livestock with insecticides/acaricides [13] (hereafter referred globally as ‘insecticides’ for simplicity). This strategy has since long been effectively used to control ectoparasites and the diseases they transmit to animals (and often also to humans), as well as to reduce the direct economic losses they cause due to decrease in productivity (e.g. lower efficiency of feed conversion, weight gain and milk production) [31]. Namely, insecticide treatment of livestock has been applied against tsetse flies transmitted animal and human trypanosomiasis in sub-Saharan Africa [32], [33], [34], [35], tick-borne diseases worldwide (such as anaplasmosis, babesiosis, theileriosis) [33], [36], and a variety of other biting and/or nuisance flies [37], [38], mosquitoes [38], [39], biting midges [40], mites, and lice.

The effectiveness of insecticide-treated livestock (ITL) against malaria was successfully tested by a community-randomised trial in Pakistan [41], where the main vectors, *An. stephensi* and *An. culicifacies*, are highly zoophilic [42]. Notably, following the treatment of virtually all domestic animals (93% of the population of cattle, sheep and goats) with a solution of the pyrethroid deltamethrin applied by sponging, *Plasmodium falciparum* malaria incidence decreased by 56% (95% CI 14%–78%), and prevalence decreased by 54% (95% CI 30–69%). Moreover, efficacy was comparable to that of traditional indoor insecticide spraying but with 80% less costs. Livestock previously infested with ectoparasites also improved in weight and milk yield productivity, enhancing community uptake of the programme [41]. Additional studies have followed to explore whether this strategy could also be applied in sub-Saharan Africa, for integrated control of malaria and animal trypanosomiasis and tick-borne diseases. Notably, bioassays of deltamethrin applied by spot-on and by spray have been conducted in Ethiopia [43], and in Tanzania [44], respectively, to assess the effects of ITL on the mortality and behaviour of malaria vectors. However, despite the encouraging results from these bioassays, the impact of ITL on malaria transmission at the community level is yet to be formally assessed in Africa, where the disease burden is the greatest, but the dynamics and determinants of infection differ from Asia.

A possible concern with ITL is repellency of mosquitoes, which may increase vector feeding on untreated livestock or unprotected humans, and make the intervention detrimental. It is known that certain insecticides exert not only (1) a toxic or direct insecticidal effect, killing mosquitoes that contact with an insecticide-impregnated surface, but also (2) behavioural avoidance responses. These sub-lethal behavioural effects include a) contact-mediated irritancy, inhibiting mosquitoes from remaining on the treated surface, thereby stimulating them to exit prematurely (common with pyrethroid insecticides), and b) non-contact or spatial repellency, which acts from a distance of the treated surface inhibiting mosquitoes from entering treated areas [45], [46]. Hereafter, the latter two responses will be referred together as repellency, since any of them could cause mosquitoes diversion to another host, in analogy with the shift in host feeding from humans to domestic animals that has occasionally been associated with the use of pyrethroid-treated nets [47], [48], [49], [50], [51]. Additionally, a case-control study in the Pokot territory of Kenya and Uganda [52] found that people with ITL had a higher risk of Visceral Leishmaniasis, suggesting that the insecticide might have repelled sandflies attempting to feed on animals and diverted them to feed on humans. Although, to the best of our knowledge, such behavioural shift has not been reported for ITL and anopheline mosquitoes, the possibility of it occurring should not be disregarded and is therefore important to investigate, particularly because the most promising insecticides tested on livestock to target malaria vectors have been pyrethroids [19], [39], [43], [44], [53], [54], [55]. The popularity of pyrethroids is due to their high insecticidal action associated with low mammalian toxicity [56], [57] which makes them safe for both the treated animals and for the consumers of animal products.

An additional concern with using ITL against malaria in Africa is that, even in areas where the moderately zoophilic *An. arabiensis* vector (which can easily feed on humans or livestock, depending on host abundance and accessibility) predominates over more anthropophilic vectors such as *An. gambiae s.s.*, the ITL intervention is still likely to achieve a smaller reduction in malaria transmission than in Pakistan (and other areas of South Asia), where the vectors are highly zoophilic, taking most of their bloodmeals upon livestock. A possible way to overcome this problem could be to artificially increase the attractiveness of insecticide-treated animals to the malaria vector. Although such has not been tested in the field yet, the use of synthetic attractants to lure anopheline vectors towards baits or traps and away from humans is an area of increasing research [58], [59].

This work aims to clarify the different effects of livestock on malaria and to understand under what circumstances livestock-based interventions could play a role in malaria control programmes. This was achieved by, firstly, developing a mathematical model that predicts the apparently contradictory outcomes that have been associated with the presence of untreated livestock in different ecological settings, and secondly, by expanding the model to incorporate insecticide treatment of livestock and fitting it to data from Pakistan (where the ITL trial was performed [41]) and from Ethiopia (where a field study was conducted [9]) to investigate the potential and limitations of ITL. We focus on livestock-based interventions, without comparing their effect with other malaria control interventions, such as insecticide-treated bednets and indoor spraying with residual insecticides. The model characterizes situations where livestock by itself can lead to a decrease, increase, or no net impact on malaria transmission to humans, and it further indicates that treating livestock with insecticide can be a useful complementary tool to control malaria, not only in Asia, but also in sub-Saharan Africa.

## Materials and Methods

### Malaria model

**Figure 1:**
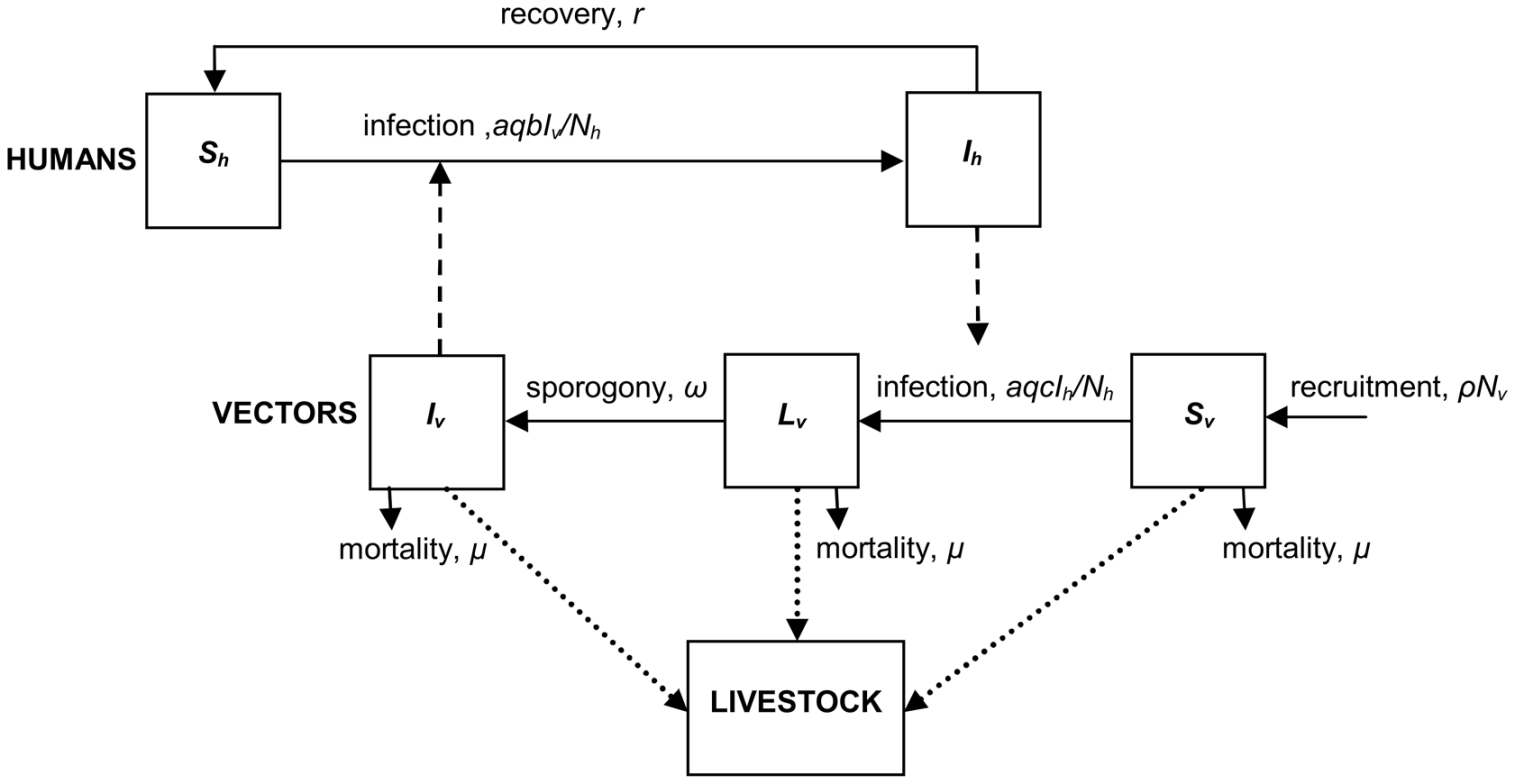
Schematic representation of the malaria model. Horizontal solid lines denote transitions between epidemiological states, and dashed lines represent transmission of infection between human hosts and mosquito vectors. Dotted lines denote vectors feeding on livestock. The vector population consists of adult female anopheline mosquitoes.

A mathematical model for the transmission dynamics of human malaria was developed based on the Ross and Macdonald models [60], [61], where humans are compartmentalized into either susceptible (uninfected and not immune), or infected/infectious (SIS model), and mosquito vectors are divided into susceptible (uninfected and not immune), exposed/latent (have been infected but are not yet infectious) or infectious (SEI model). Here, the Ross-Macdonald model is extended by discriminating the feeding behaviour of the vector on its alternative hosts: livestock and human populations, and by incorporating the treatment of livestock with insecticide as a potential *novel* method to control human malaria. The new model explicitly incorporates the effects of untreated and insecticide treated livestock on the vector population feeding behaviour, mortality and population density, allowing exploration of the impact of livestock-based interventions on malaria transmission dynamics. A diagrammatic flow chart of the model is presented in Figure 1. Throughout the article, the human, vector and livestock populations will be referred to with the subscripts *h*, *v* and *l*, respectively.

The model is formally represented by a system of ordinary differential equations as follows. For the dynamics of infection in the human population, we have
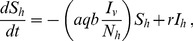
(1)

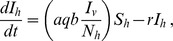
where *N_h_ = S_h_+I_h_* (total human population). Transmission of infection from vectors to humans depends on the number of infected vectors per human, *I_v_/N_h_*, the vector blood feeding rate on any host, *a*, (the interval between bloodmeals on any host is 1/*a*), the proportion *q* of feeds taken on humans (so-called human blood index - HBI), the probability *b* that a human will become infected following the bite of an infectious vector, and the number of susceptible hosts, *S_h_*. Once susceptible humans are infected the parasite undergoes a period of latency before infective gametocytes appear, but as this period is short compared to the duration of infection, it is not represented explicitly in the model [62]. Infected individuals, *I_h_,* recover from infection at a rate *r*, eventually becoming fully susceptible to re-infection (the average duration of infection is 1/*r*). It is therefore assumed that there is no boosting immunity due to repeated infections, as done for simplification in earlier zooprophylaxis models [27], [63], [64], [65], [66]. Human natural mortality and reproductive rates are omitted from the model because humans have a long life expectancy relative to other time periods used in the model (such as the latent period, infectious period and vector life span). We also assume no disease-induced death and therefore, the human population size remains constant.

The disease dynamics in the vector population is represented by
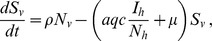


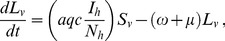
(2)

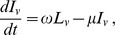
where *N_v_ = S_v_+L_v_+I_v_* (total vector population). The vector population comprises only adult female anopheline mosquitoes, since males do not blood feed. Transmission of infection from humans to vectors depends on the proportion of infectious humans, *I_h_/N_h_,* the vector feeding rate on humans, *aq*, and the probability *c* that a vector will become infected after feeding upon an infectious human. Infected latent mosquitoes, *L_v_*, become infectious after a sporozoite maturation period (latent period  = 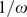). Anopheline vectors are assumed to remain infectious throughout their life, as usually observed. Infection is assumed to have no impact on vector feeding behaviour, reproduction, nor mortality, as in most malaria models. Although some effects from infection have been described [67], they are not considered in the approximation adopted here.

The vector life expectancy is often about the same order of magnitude as the latent period in the vector. Consequently, only a minority of the infected vector population survives to become infectious, and therefore the model must incorporate the class of latent vectors as well as vector mortality and recruitment. The mortality rate of adult vectors, 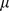, is assumed to be age independent, such that the average vector life-span is 1/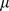. We consider two implementations for the recruitment rate. One where the vector population is kept constant by assuming that recruitment and mortality rates are equal 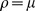.

Another where the density-dependent regulation of the adult vector population due to competition within the larval stages, which depends on the abundance and extent of breeding sites, is explicitly modelled. Following Lord et al. [68] and Kawaguchi et al. [66], the recruitment rate of newly emerged female adults entering the susceptible class is given by
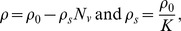


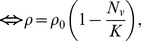
where 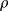 and 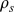 are the vector recruitment rate in the absence of density-dependence constraints and the strength of the density-dependence in recruitment, respectively, and *K* is the vector-carrying capacity of the ecosystem. It is assumed that the number and capacity of the breeding sites (and therefore, the vector-carrying capacity) remain the same independently of the hosts' abundance and availability. For instance, the potential increase in breeding sites due to livestock hoof prints is not considered here. While density dependence is essential for the systematic investigation of the zooprophylactic effects of livestock populations with different sizes and characteristics, this is no longer a focus on the investigation of insecticide treatment later on. As such, variable vector population is primarily used in the first part of the Results (Untreated Livestock) and the constant vector population is the implementation of choice throughout the second part (Insecticide Treated Livestock).

As done for simplification in previous malaria models, we assume that vectors take one bloodmeal per gonotrophic cycle, and therefore, the interval between bloodmeals corresponds to the length of the gonotrophic cycle. Similarly, female mosquitoes are assumed to feed homogenously with a fixed preference for humans and/or animals.

### Formulation of livestock effects

In the absence of insecticide treatment, the effects of livestock on the human blood index, *q*, follow what has been proposed by Sota and Mogi [63] and are defined by:
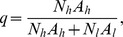
which can be simplified to:



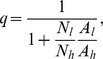
where *A_h_* and *A_l_* are the proportional availabilities of the human and livestock hosts, respectively, and can take any value between 0 and 1, inclusive. The term availability encompasses all the factors that can influence the likelihood of the vector feeding on a given type of host, when two types of alternative hosts are present in equal numbers. Namely, these factors include the accessibility of each host to the vector (which can vary with distance between vector breeding sites and location of humans/livestock at night, whether located indoors or outdoors, under a bednet or not, or livestock enclosed inside a shed or not), and on the intrinsic propensity to feed upon humans *versus* animals (anthropophily *versus* zoophily), and to feed in the location where the host resides (endophagy *versus* exophagy), which can be modified by vector genetics and learning. In the presence of insecticide treatment, the expression for the human blood index is generalized as
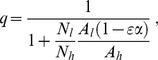
(3)where 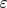 is the proportion of livestock population treated with insecticide, hereafter referred as treatment coverage, and 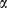 is the diversion probability, defined as the probability that a host-seeking mosquito will be diverted away from (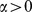, repellency) or towards (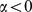, attractancy) an insecticide-treated animal. Therefore, the insecticide treatment of livestock only affects the human blood index if the intervention has some diversion effect upon the vectors, either repellency or attractancy (see **Text S1.1** for more details).

The baseline mortality rate of the vector is decomposed as being the sum of the minimum mortality rate (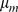) due to causes other than searching for a bloodmeal host (i.e. mortality due to hazards during the act of feeding on a host, the gestation period, the search for oviposition sites, and the underlying aging process), and the mortality due to searching for a bloodmeal host (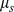). The search-related mortality is assumed to be proportional to the length of the searching period, which is inversely related to the abundance and availability of potential blood meal hosts. These assumptions follow previous models by Saul [27] and Killeen and Smith [65].

When no livestock are treated with insecticide, the expression for the vector mortality rate therefore becomes
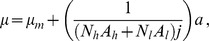
where the last term is the search-related mortality, 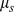. Parameter *j* is a factor to scale the proportional availabilities (*A_h_*, *A_l_*) of hosts to the mosquito vectors into absolute availability values. As in most previous malaria models, it is assumed that the feeding success of malaria vectors is independent of the density of vectors per available host [69].

When livestock are treated with insecticide the mortality rate is generalized as
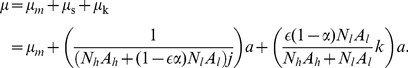
(4)


In the 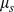 term, as seen for the human blood index, if there is repellency (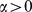) it is as if the availability of livestock became reduced by the proportion 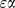, which corresponds to the proportion of bites attempted on a given animal that will be diverted to another animal or human host. This will cause an increase in the time it takes for the vector to find a bloodmeal host, with consequent increase in the search-related vector mortality. Conversely, if there is attractancy (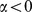), it is as if the availability of livestock became increased by the proportion 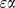, which corresponds to the proportion of bites attempted on a given animal that were diverted from another animal or human host. This will decrease the time for the vector to find a bloodmeal host, thereby decreasing the search-related vector mortality. The 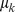 term accounts for the direct lethal effect of insecticide applied on livestock, and is a function of the vector biting rate on livestock, the treatment coverage, 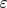, the diversion probability, 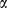, and the insecticidal probability, *k*. The daily biting rate, *a*, needs to be included in the expressions for 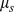 and 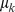, since the additional mortalities, either due to searching for a bloodmeal host or due to attempting to feed on insecticide-treated livestock, are only suffered by the vector when it attempts to blood feed (see **Text S1.2** for more details). Our model assumes that the insecticide effects (diversion and insecticidal probabilities) are constant, therefore reflecting average values of what would be observed throughout the year.

### Simulations

The system of equations (1)–(2) was analysed symbolically for the derivation of endemic equilibrium solutions (see **Text S2**) and numerically for the simulation of dynamical trajectories over time. Numerical integration was performed using BERKELEY MADONNA v. 8.3.9, with the built-in method *fourth order Runge-Kutta*. The equilibrium solutions were further explored with MATLAB v. R2011a.

We first investigate the effects of untreated livestock in malaria transmission and then move to explore the impact of treating livestock with an insecticide that has lethal and possible diversionary effects (repellency or attractancy) upon malaria vectors. For this purpose, a range of simulations was performed with system (1)–(2), focusing on scenarios of endemic *Plasmodium falciparum* malaria.

### Threshold derivation

We also determined the threshold conditions required for persistence of malaria, by analyzing the equilibria of the model represented by system (1)–(2). The average number of secondary cases generated by a single infectious individual introduced in a population of fully susceptible individuals, is known as the basic reproduction number, denoted by *R*_0_ [61], [70]. This threshold quantity expresses the transmission potential of an infectious disease and must exceed unity for the infection to be maintained in the population. The expression for *R*_0_ was derived by linearization around the disease-free equilibrium (DFE), based on the next-generation operator approach [71], [72]. We then explored the impact of ITL on *R*_0_ for different intervention scenarios. Namely, by setting *R*_0_ = 1, we obtained the critical proportion of the livestock population that must be treated with insecticide, and assessed how this critical coverage would be affected by the insecticide diversionary properties.

### Parameterization

Parameters values for the untreated livestock model were obtained directly or derived from the literature and are provided in Table 1. The effects of insecticide treatment were explored using parameter values that were either extracted or derived from empirical data from the index studies in the North-West Frontier Province of Pakistan (ITL trial conducted by Rowland et al. [41]) and in the Konso district of South-West Ethiopia (field study by Franco [9]), or from previous studies within or near the area of the index studies, as listed in Table 2. See **Text S3** for details on parameterization.

**Table 1:** Parameter values for modelling the effects of untreated livestock on malaria.

Symbol	Definition	Value	[Reference]
*a*	Vector daily biting rate on any host	0.5	[89]
*b*	Probability that humans become infected from the bite of an infectious vector	0.04	[90]
*c*	Probability that vectors become infected after biting on an infectious human	0.3	[90]
*r*	Human daily recovery rate from infection (1/average duration of infection)	0.05	[29], [30], [90]
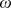	Daily rate at which infected mosquitoes become infectious (1/latent period)	0.07	[91]
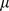	Overall average vector daily mortality rate 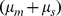	Varied	Derived
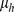	Vector daily mortality rate in absence of available livestock	0.1	[89]
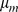	Vector daily minimum mortality rate when there are no hazards due to search for a bloodmeal host	0.05**	[89]
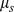	Vector daily mortality rate due to searching for a bloodmeal host	Varied**	Derived
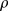	Overall average vector daily recruitment rate	Varied	Derived*
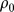	Vector daily recruitment rate in the absence of density-dependence constraints	Varied	Derived*
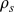	Strength of the density-dependence in recruitment (/day)	Varied	Derived*
*K*	Carrying capacity of the vector population (/ha)	10^3^ to 10^5^	-
*N_v_*(0)	Initial vector density, prior to change in livestock abundance and/or availability (/ha)	10^3^	-
*N_h_*	Human density (/ha)	100	-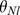	Relative density of livestock:humans (*N_l_*/*N_h_*)	0 to 20	-
*A_l_*	Proportional availability of livestock to vectors	0 to 1	-
*A_h_*	Proportional availability of humans to vectors ( = 1-*A_l_*)	0 to 1	-
*q*	Proportion of vector bloodmeals on humans (Human Blood Index)	0 to 1	Derived
*j*	Scaling factor to transform proportional availabilities into absolute availabilities	Varied	Derived

*For simulations with constant vector population density: 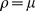; for variable vector density: 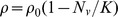 and 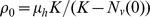.

**The relative magnitudes of 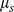 and 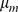 were varied in a sensitivity analysis.

**Table 2:** Parameter values for modelling the effects of insecticide-treated livestock on malaria.

Symbol	Definition	Value		[Reference]	
		Pakistan	Ethiopia	Pakistan	Ethiopia
*a*	Vector daily biting rate on any host (1/gonotrophic cycle)	0.4	0.4	[92]	[93], [94]
*b*	Probability that humans become infected from the bite of an infectious vector	0.5	0.5	[95], [96]	[95], [96]
*c*	Probability that vectors become infected after biting on an infectious human	0.95	0.07	**	**
*r*	Human daily recovery rate from infection (1/average duration of infection)	0.05	0.05	***	***
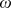	Daily rate at which infected mosquitoes become infectious (1/latent period)	0.057	0.064	Derived from [9], [91]	Derived from [9], [91]
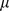	Overall average vector daily mortality rate 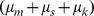	Varied	Varied	Derived	Derived
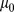	Vector daily natural mortality rate in the absence of ITL (1/natural life expectancy)	0.22	0.12	Derived from [41], [92]	Derived from [93], [94], [97]
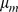	Vector daily minimum mortality rate when there are no hazards due to search for a bloodmeal host (1/vector maximum life expectancy)	0.11****	0.06****	-	-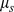	Vector daily mortality due to searching for a bloodmeal host*	0.11****	0.06****	Derived	Derived
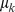	Vector daily mortality due to the direct lethal effect of insecticide applied on livestock	Varied	Varied	Derived	Derived
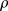	Overall average vector daily recruitment rate	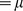	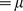	-	-
*N_v_*	Vector density (/ha)	5000	1500	**	**
*N_h_*	Human density (/ha)	100	100	-	-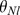	Relative density of livestock:humans (*N_l_*/*N_h_*)	0.14	1.13	[41]	[9]
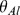	Relative availability of livestock:humans (*A_l_*/*A_h_*)	53.24	0.938	Derived from [98]	Derived from [99]
*A_l_*	Proportional availability of livestock to vectors 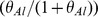	0.982	0.484	Derived	Derived
*A_h_*	Proportional availability of humans to vectors (1-*A_l_*)	0.018	0.516	Derived	Derived
*q*	Proportion of vector bloodmeals on humans*	0.118	0.485	Derived	Derived
*j*	Scaling factor to transform proportional availabilities into absolute availabilities	Varied	Varied	Derived	Derived
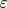	Treatment coverage: proportion of livestock population that is treated with insecticide	0 to 1	0 to 1	-	-
*k*	Insecticidal probability	0.1 (0 to 0.9)	0.1 (0 to 0.9)	Derived from [54]	-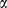	Diversion probability ( 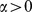 , repellency; 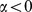 , attractancy)	0 to 1	−1 to 1	-	-

Malaria vectors: *An. culicifacies* in Pakistan, *An. arabiensis* in Ethiopia.

*Parameter values pre-intervention that will be affected if livestock are treated with an insecticide with diversion properties.

**Values chosen to produce malaria prevalence similar to the observed in the index study areas.

***M. Rowland unpublished data.

****The relative magnitudes of 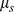 and 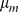 were varied in a sensitivity analysis.

## Results

### Untreated livestock

Here we explore the effects that varying the abundance and/or availability untreated livestock could have on different outcome measures of malaria transmission. All the simulations used parameter values as listed in Table 1, unless otherwise specified.

**Figure 2:**
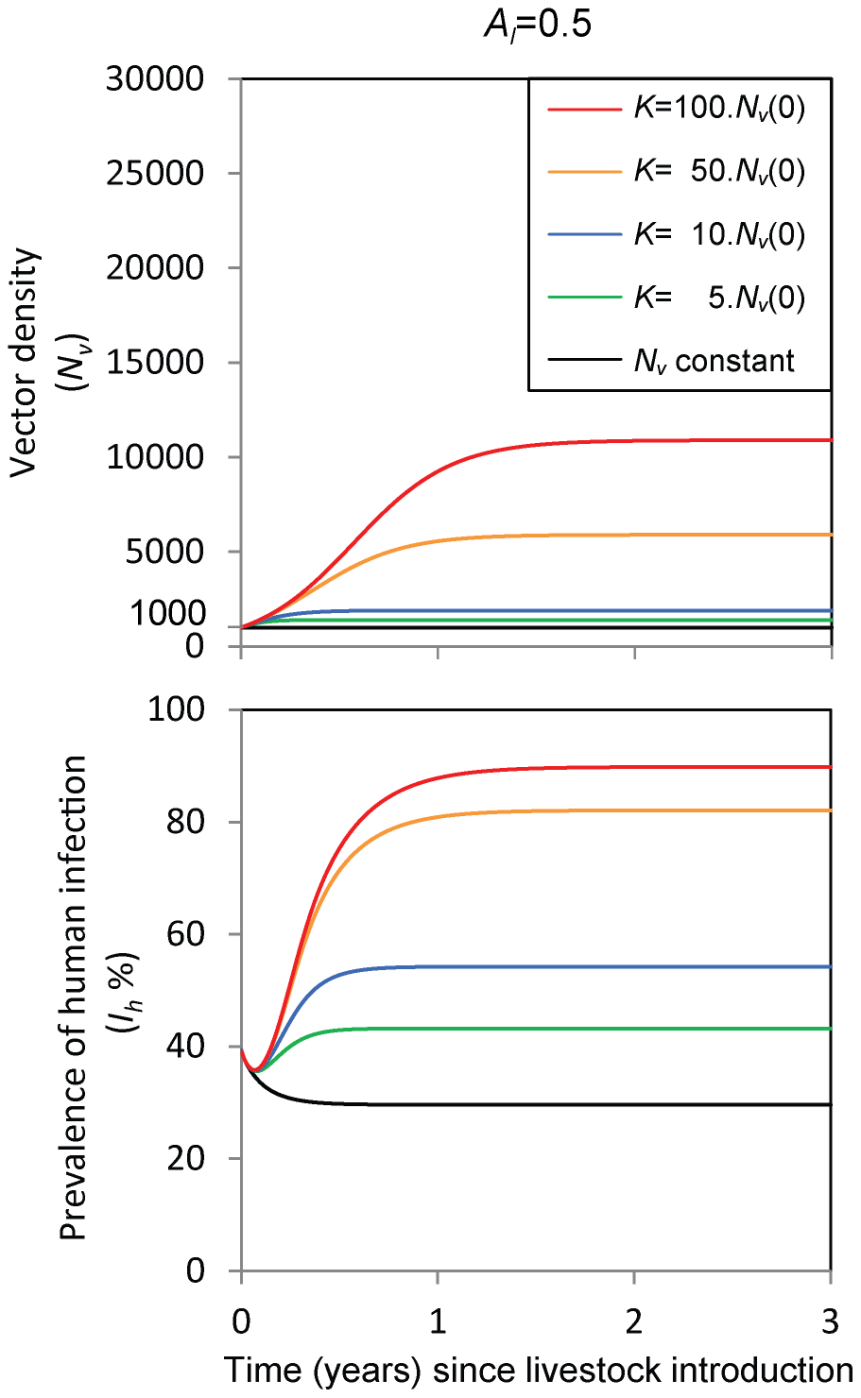
Temporal effect of introducing livestock in a setting with endemic malaria. Effect of introducing livestock in a setting where only humans were present, when: *N_v_* remains constant (black line), and when *N_v_* increases until reaching a maximum, which depends on the carrying capacity, *K* (increasing from *K* = 5,000 (green) to 100,000 (red)). *N_v_*(0) = 1000, *N_h_* = 100 and *A_l_* = 0.5: the availability of livestock to vectors is the same as that of humans; 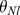 = 0.25 (1 head of livestock per 4 persons). To achieve the same initial equilibrium *N_v_* (and *I_h_*) for various *K* values, the vector recruitment rate in the absence of density-dependence constraints was set to vary accordingly: 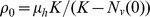. Other parameters are as in Table 1.

**Figure 2** shows the vector population density and prevalence of human infection over time as livestock are introduced in a setting where previously only humans and no livestock were present. Simulations were performed assuming a fix human density (*N_h_* = 100) and 1 head of livestock per person (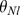 = 0.25). The proportional availability of livestock to vectors was the same as that of humans for all plots in this figure (*A_l_* = 0.5), illustrating the case of a moderately zoophilic vector, like *An. arabiensis* in Ethiopia. Additional scenarios of host density and availability were also explored.

Firstly, we simulate a modified model with the best case scenario where the vector population is kept constant (*N_v_*(t) = 1000) by assuming that recruitment and mortality rates are the same (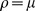) (black line), and secondly, the carrying capacity was set to a higher level (*K* = 5,000 to 100,000) and the vector population density increased from its initial equilibrium (*N_v_*(0) = 1000) towards carrying capacity (coloured lines). As we would expect, in the case of constant vector density, the introduction of livestock leads to consistent reductions in the prevalence of human cases by diverting vector feeds to livestock (black line). Overall, the higher the numbers and/or availability of the introduced livestock, the stronger is the predicted zooprophylactic effect on malaria transmission. When the vector population density is allowed to increase, however, the prevalence of human cases might increase (coloured lines). For a given density and availability of livestock, the higher the carrying capacity is in relation to the initial vector population density, the higher the vector density and consequently malaria transmission levels in the new endemic equilibrium. In all simulated scenarios in **Figure 2** the system reaches a new equilibrium in less than 3 years after the introduction of livestock.

**Figure 3:**
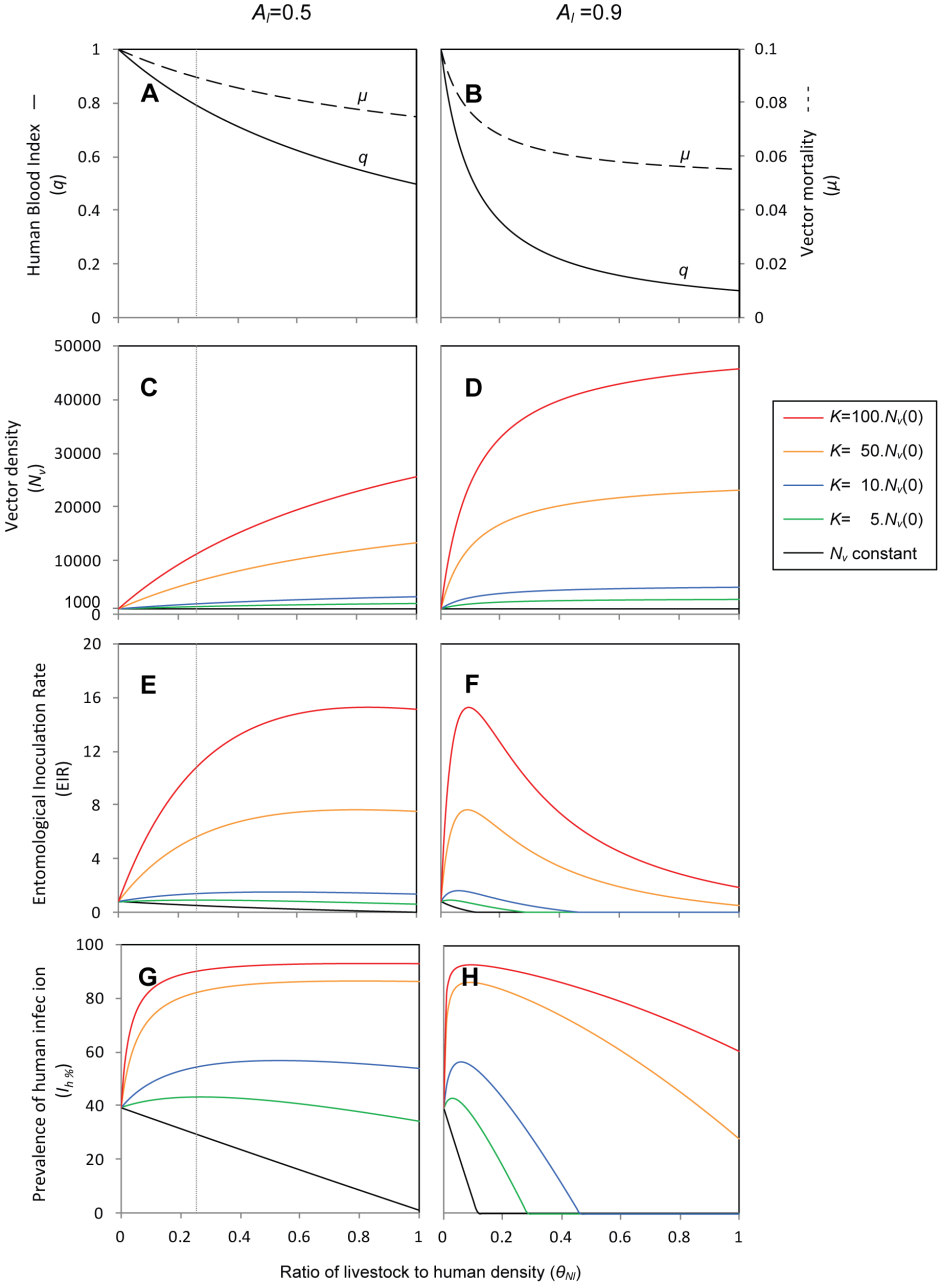
Effect of altering the relative livestock to human density, for different vector density scenarios, at the new endemic equilibrium. Comparing a scenario where the availability of livestock to vectors is the same as that of humans (left, *A_l_* = 0.5) *versus* where it is 9 times higher than that of humans (right, *A_l_* = 0.9). Along the *x*-axis, representing 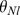 = *N_l_/N_h_*, the livestock density *N_l_* is varied relative to a fixed human density *N_h_* = 100. *N_v_*(0) = 1000. Effect of introducing livestock when: *N_v_* remains constant (black line), and when *N_v_* increases until reaching a maximum, which depends on the carrying capacity, *K* (coloured lines: *K* increasing from 5,000 (green line) to 100,000 (red line)). The effects of introducing livestock on the human blood index (HBI) and on the vector mortality rate (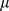) are independent from the vector density scenarios (A, B). The vertical line in the left panels highlights the new endemic equilibrium that is reached after the introduction of 1 head of livestock per 4 persons (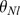 = 0.25), corresponding to the end of the timeline in Figure 2. Other parameters are as in Table 1.

**Figure 3** examines various outcome measures of malaria transmission that characterize the new endemic equilibrium that is reached under a range of relative livestock to human density (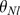 varying from 0 to 1), when the proportional availability of livestock to vectors is either the same (*A_l_* = 0.5) or nine times higher (*A_l_* = 0.9) as that of humans, the latter resembling a scenario of a highly zoophilic vector, ∼ *An. culicifacies* in Pakistan. The outcome measures investigated include the human blood index (HBI, designated as *q* in our model), daily overall vector mortality (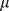), vector density (*N_v_*),daily entomological inoculation rate (EIR), and prevalence of infection in humans (*I_h_*). The EIR is the number of infective mosquito bites received by a human per unit time, estimated multiplying the daily human-biting rate (HBR) by the proportion of mosquitoes with sporozoites in their salivary glands (*I_v_*/*N_v_*). The HBR is the total number of mosquito bites received by a human, per day, and is calculated as the product of the number of vectors per human and the number of daily bites on humans per vector (HBR = (*N_v_*/*N_h_*)*a*HBI).The figure illustrates the effects of livestock on decreasing the human blood index while decreasing vector mortality (**Figure 3A,B**) and increasing vector population density (**Figure 3C,D**). The combination of these effects may lead to situations where the presence of livestock increases, decreases, or has no significant impact on malaria transmission (all other panels in **Figure 3**). The introduction of livestock is predicted to have a zooprophylactic effect, i.e. decrease malaria transmission, in two situations. One is that the vector population density does not increase as a result of livestock introduction. The other is that although the vector population density increases as a result of livestock introduction, the livestock numbers and availability to vectors are sufficiently high, such that the increase in vector density is counteracted by the diversion of bites from humans to animals (**Figure 3**). Otherwise, the introduction of livestock is predicted to increase malaria transmission.

#### Impact of vector search-related mortality on the effects of untreated livestock

For the purpose of illustrating the model behaviour, the simulations for untreated livestock assume that the vector-search mortality when no livestock are available (i.e. when *N_l_* = 0 or *A_l_* = 0), has the same value as the vector minimum mortality rate 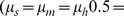
0.05/day). A sensitivity analysis was done to explore the impact of different relative magnitudes of the vector search-related mortality (**Figure S1** in **Text S4.1**). If the vector search-related mortality is already negligible before livestock are introduced, then introducing livestock will have no impact on the vector mortality, and will simply decrease HBI, consequently decreasing malaria transmission. Conversely, if the vector search-related mortality is considerable, introducing livestock can considerably decrease vector mortality, which can increase the proportion of vectors surviving the extrinsic incubation period to become infectious, and thereby counteracting the decrease of the HBI due to diversion of mosquito bites from humans to livestock, consequently increasing malaria transmission. After a certain threshold of livestock density, further increasing their abundance produces negligible reduction on vector mortality.

### Insecticide treated livestock

To explore the effects of ITL on malaria the model was fitted to *P. falciparum* malaria transmitted by the highly zoophilic *An. culicifacies* in Pakistan and the more anthropophilic *An. arabiensis* in Ethiopia. Parameter values are listed in Table 2. The main differences in the malaria transmission parameters between the Asian and African settings are as follows. In Ethiopia, livestock were 8.1 times more abundant, although with an estimated 56.8 times lower availability to the main malaria vector, than in Pakistan, resulting in a predicted HBI over 4 times higher in the African than in the Asian setting. Additionally, the estimated duration of the latent period in vectors was slightly shorter, while the vector life expectancy was 75% higher in Ethiopia than in Pakistan. The initial density of vectors per human and the probability of infection in vectors were set to be, respectively, 3.3 and 13.6 times higher in Pakistan than in Ethiopia.

It was assumed that, prior to the ITL intervention, an endemic equilibrium of malaria transmission had been reached and, as in most previous zooprophylaxis models [27], [65], [66], vector population density was at its equilibrium level, and remained constant throughout the intervention (i.e. vector recruitment and mortality rates are the same). We therefore consider the scenario where the insecticide has no impact on the overall vector population density. Thus, the beneficial impact of an ITL intervention with a non-diversionary insecticide is assumed to be due only to the decrease on vector survival caused by the toxic insecticidal effect, and consequent reduction in the proportion of vectors that become infectious. When the insecticide additionally has some repellent properties, there is some beneficial effect from increasing the vector search-related mortality, which partially counteracts the increase in vector bloodmeals on humans. Conversely, when there is attractancy, there is the greater benefit of decreasing the bloodmeals in humans, which counteracts the decrease in vector search mortality.

#### Impact on malaria prevalence

We started by exploring the predicted impact of ITL on the prevalence of human infection. This is represented in terms of the prevalence ratio (PR), which is defined as the ratio between the prevalence under a given coverage of insecticide-treated livestock (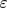) and the prevalence pre-intervention. The proportional reduction on the pre-intervention prevalence is given by 1-(prevalence ratio).

**Figure 4:**
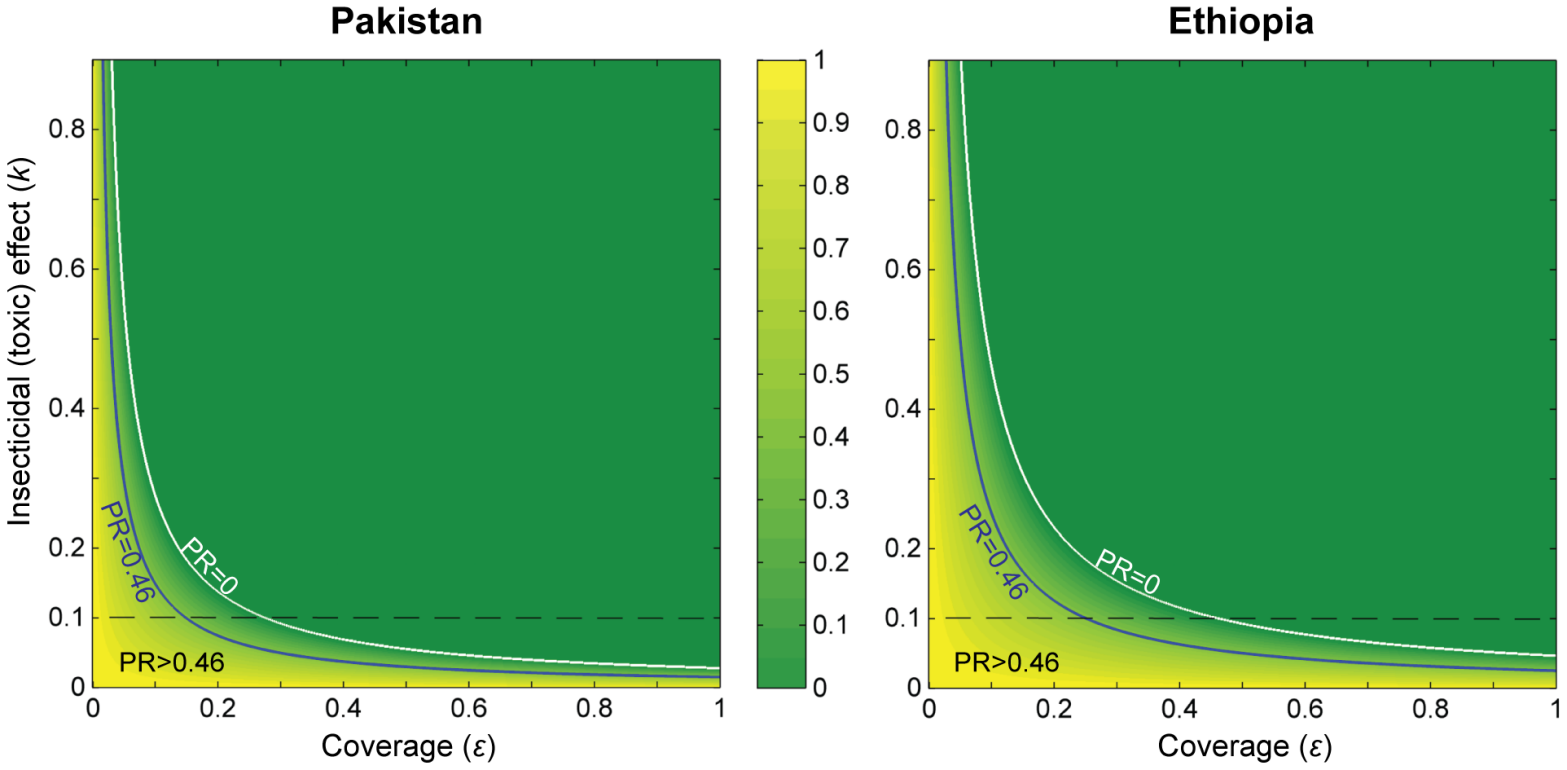
Predicted impact of Insecticide Treatment of Livestock on malaria prevalence, without diversion (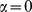). This figure shows the combination of values of coverage and insecticidal probability required to achieve a given prevalence ratio (PR: prevalence with ITL / baseline prevalence). Blue line: PR = 0.46 (like the observed in the Pakistan trial); White line: PR = 0; Dashed line: *k* = 0.1, as estimated for the Pakistan trial. The colour bar shows the scale of PR values, from 0 to 1. Other parameters are as in Table 2.

Simulations were initially performed to estimate the coverage of treated livestock (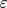) and insecticidal probability (*k*) required to obtain the 54% reduction in *P. falciparum* prevalence observed in the Pakistan ITL trial, for the Pakistan and the Ethiopian simulated scenarios, assuming the use of an insecticide with no diversion properties (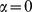, **Figure 4**). For any given intervention effort, ITL is predicted to cause a stronger reduction in malaria prevalence in Pakistan than in Ethiopia. Nevertheless, the same reduction in prevalence could be achieved in Ethiopia, if using higher treatment coverage and/or a product with stronger or longer lasting insecticidal properties. For instance, for the scenario of *k* = 0.1 (estimated value from Pakistan data, as detailed in **Text S3.2**), the predicted treatment coverage required to obtain the observed reduction in prevalence (PR = 0.46) is 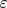 = 15% in Pakistan and 25% in Ethiopia (**Figure 4**).

**Figure 5:**
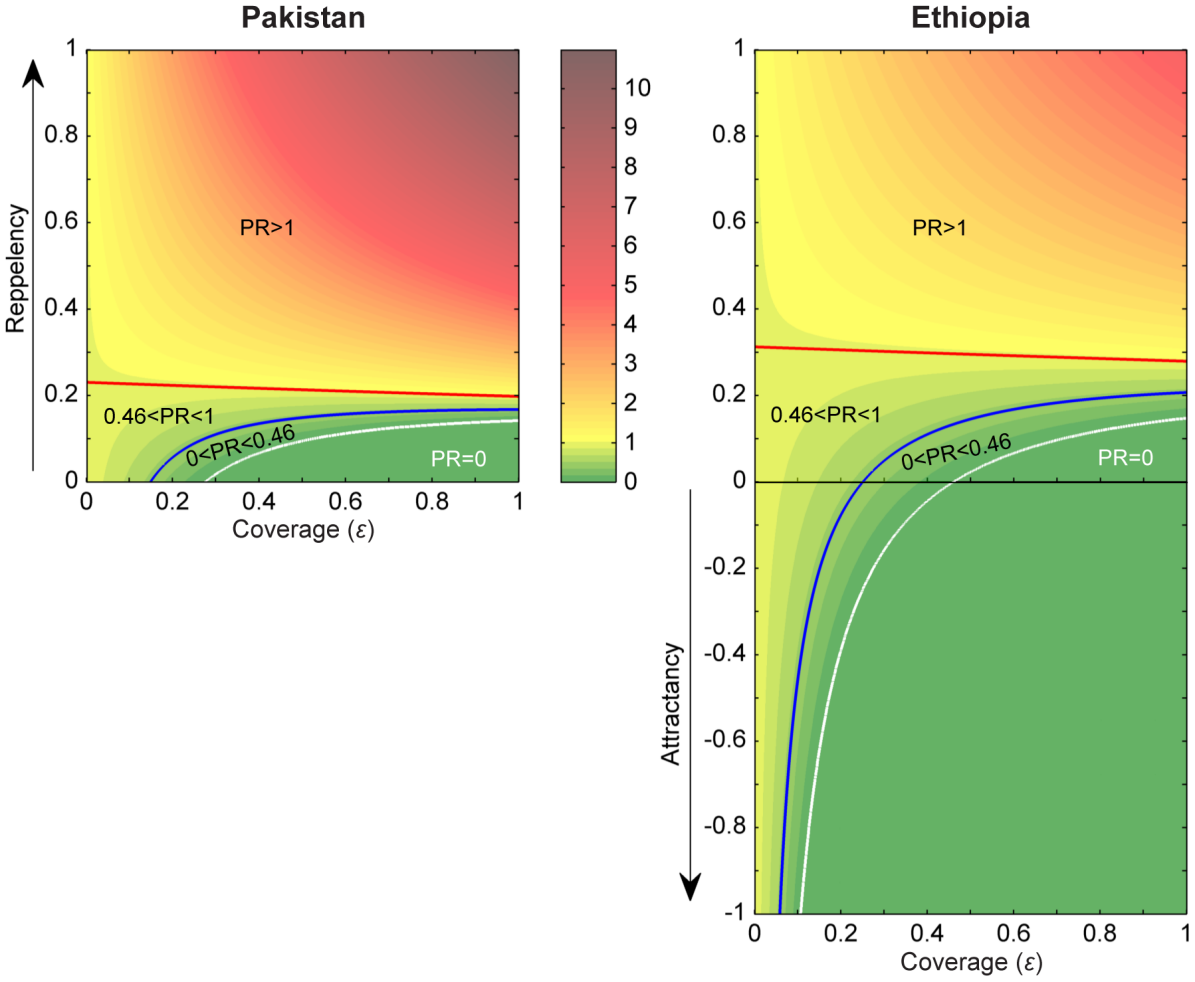
Predicted impact of Insecticide Treatment of Livestock on malaria prevalence – with repellency (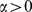) or attractancy (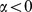) for *k* = 0.1. This figure shows how the diversionary properties of the insecticide affect the coverage required to achieve a given prevalence ratio (PR: prevalence with ITL / baseline prevalence). Blue line: PR = 0.46 (like the observed in the Pakistan trial); White line: PR = 0; Red line: PR = 1 (above which treating livestock increases malaria prevalence). Along the y axis, 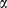 is varying from no diversion (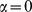) to maximum repellency (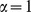) or maximum attractancy (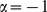). The colour bar shows the scale of PR values, from 0 to ≈11 in Pakistan and up to ≈5 in Ethiopia. Other parameters are as in Table 2.

We also investigated whether by increasing the attractiveness of insecticide treated livestock to vectors it would be possible to obtain in Ethiopia the same reduction in prevalence as observed in the Pakistan trial, with the same intervention effort used in the Asian setting (**Figure 5**). To achieve in Ethiopia the same PR = 0.46 with similar coverage as predicted for Pakistan (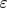 = 15%, for *k* = 0.1, assuming no repellency) would require an attractancy of 20%, while with attractancy of 10% or 30%, the coverage would be approximately 19% or 13%, respectively.

We then explored how the coverage would be affected if the insecticide had a repellency effect upon vectors, and what might be the repellency probability above which the intervention could become deleterious, by causing prevalence to increase above the pre-intervention level. Not surprisingly, the intervention benefits considerably decrease if the insecticide has repellency properties. Considering again the case of the estimated *k* = 0.1 (**Figure 5**), to achieve the observed reduction in prevalence (PR = 0.46) with the 93% coverage that was actually applied in the Pakistan trial, the model suggests that a repellency probability of ∼17% would need to be acting in Pakistan. If that same repellency level was acting in Ethiopia, the required coverage was predicted to be 60%. For repellency above 17% in Pakistan or above 21% in Ethiopia, the achieved reduction in prevalence is expected to be always smaller than the observed (i.e. the prevalence ratio, PR, would always be >0.46), even if all livestock are treated (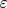 = 1). The intervention would become deleterious (PR >1) for repellency above 20% in Pakistan and above 28% in Ethiopia (**Figure 5**). The smaller the coverage (for a given *k*), or the greater the *k* (for a given coverage), the higher is the repellence threshold above which ITL will start becoming detrimental (PR >1) (**Figure S2** in **Text** **S4.2**).

#### Threshold phenomena

The derived basic reproduction number for the malaria model is given by:
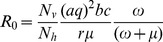
(5)where *q* is given by expression (3) and 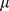 is given by expression (4).

By setting *R*_0_ = 1 in (5) we see that the critical proportion of the livestock population that must be treated with insecticide in order to interrupt malaria transmission is
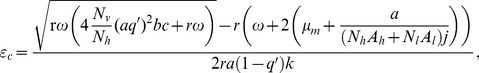
(6)where 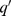 is the HBI in the absence of insecticide treatment: 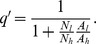


This expression for 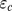 is valid for the best-case scenario regarding repellence and the worst case scenario regarding attractancy, i.e. when using an insecticide without any diversionary effects upon vectors (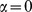).

**Figure 6:**
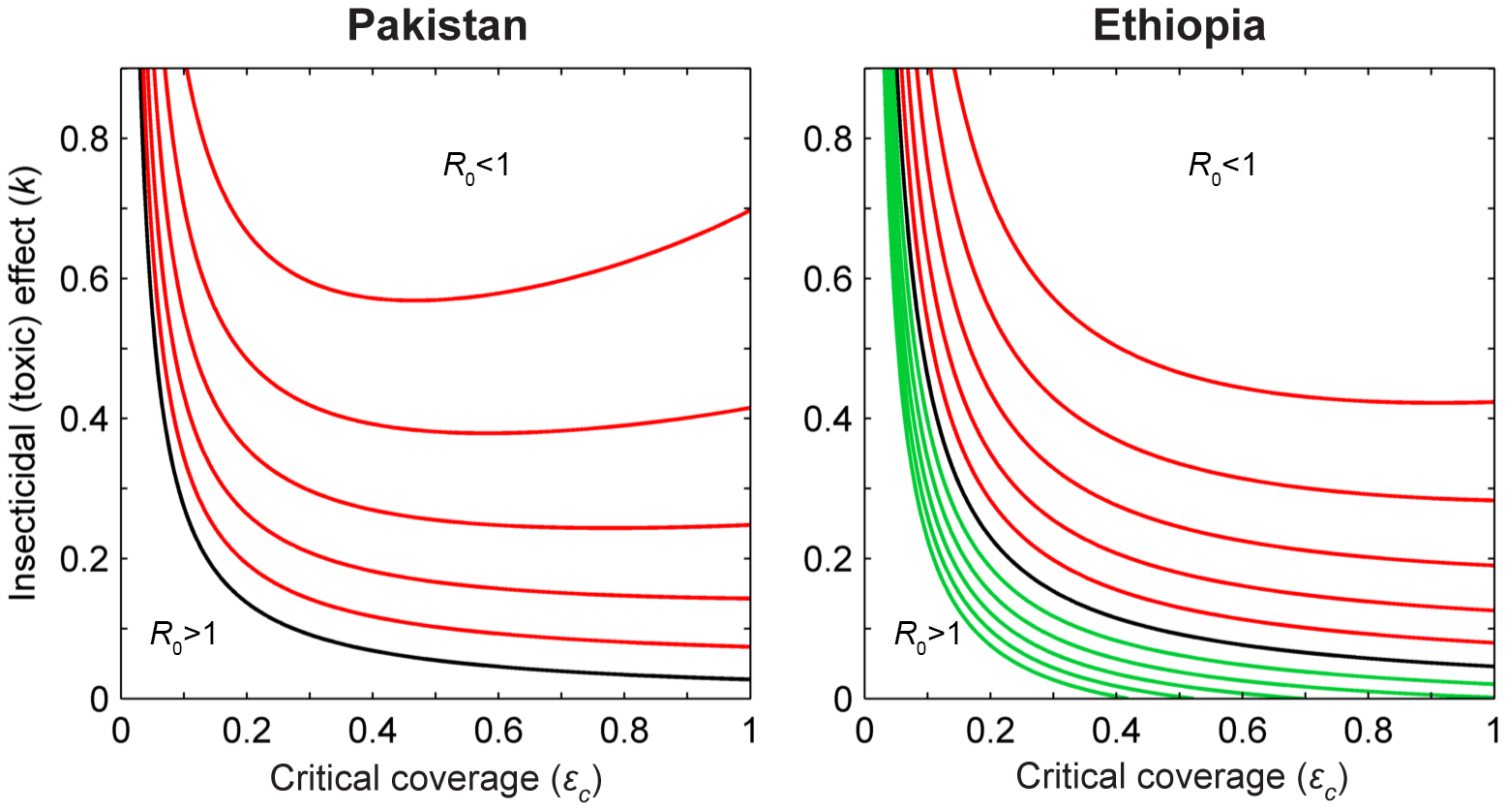
Critical proportion of ITL as a function of the insecticidal (*k*), and diversionnary effect (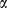). The lines show the combination of values of coverage and insecticidal probability required to achieve *R*_0_ = 1, above which *R*_0_ will be decreased below 1, for a given diversion probability (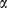). Black line: 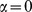, no repellency or attractancy (is the same as the white line in Figure 4); Red lines: 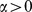, repellency increasing from 0.1 to 0.5 (top), at intervals of 0.1; Green lines: 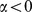, attractancy increasing from −0.1 to −0.5 (bottom), at intervals of 0.1. Other parameters are as in baseline simulations (Table 2).

To explore how repellency (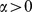) or attractancy (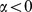) would impact 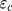 numerical simulations were performed (**Figure 6**). The stronger the insecticidal probability (*k*), the smaller is the critical proportion of treated livestock (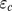) required to potentially reduce *R*_0_ below unity, for any given 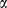 in Ethiopia, and for 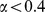 in Pakistan. In the Asian scenario, for 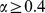, above a certain treatment coverage and insecticidal probability, there could be a shift from *R*_0_<1 to *R*_0_>1. For instance, for 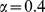 and *k* = 0.4, *R*_0_ becomes less than 1 if coverage is above 36% and below 90%, while for coverage above 90% then *R_0_* increases to greater than 1. Similarly, for 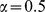 and *k* = 0.6, *R*_0_ is reduced to less than 1 for coverage between 29% and 70%, but for coverage above 70% the *R*_0_ becomes above 1. For any given *k*, the stronger the repellence, the higher is the critical coverage, while the stronger the attractancy, the lower is the critical coverage. Furthermore, for any given *k*, with or without repellency, the critical coverage is always higher for Ethiopia than for Pakistan (**Figure 6**).

#### Impact of vector search-related mortality on the effects of insecticide-treated livestock

The baseline simulations for ITL assume that the background vector search-related mortality (pre-livestock treatment) has the same value as the vector minimum mortality rate 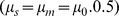. Additional simulations were done to explore the sensitivity of the findings to alternative search-related vector mortality values (**Figure 5** and **Figure 6** can be contrasted with **Figure S3** and **Figure S4** in **Text S4.2.1**, respectively).

Although there is uncertainty about its exact value, the relative magnitude of the background vector search-related mortality will only affect the intervention impact if the insecticide has diversionary properties. Namely, decreases in the background search mortality will counteract the only benefit of repellence (which was an increase on the search-associated vector mortality), and consequently decrease the beneficial impact of an ITL intervention. In general, the smaller the background search-related mortality, the stronger is the detrimental effect of any given repellency probability (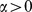) on malaria prevalence or *R*_0_, and consequently, the greater is the coverage required to achieve a given reduction in prevalence or *R*_0_, and the lower is the repellence threshold above which the intervention would become deleterious (and *vice-versa*). For instance, comparing the baseline scenario with the worst-case scenario of null background vector search-related mortality, the repellence threshold would decrease from 20% to 13% in Pakistan and from 28% to 19% in Ethiopia (**Figure S3** in **Text S4**). This relationship becomes however increasingly non-linear with increase in the insecticidal effect (*k*) of a treatment with repellency, namely in Pakistan (**Figure S4** in **Text S4**). For a given attractancy probability (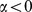), the smaller the background vector-search related mortality, the stronger are the intervention benefits, and consequently, the smaller is the coverage required to achieve a given reduction in prevalence or *R*_0_ (**Figure S3** and **Figure S4** in **Text S4**).

## Discussion

By combining a mathematical model with field data we have explored the different effects that livestock can have on human malaria in areas where the disease is transmitted by zoophilic vectors, allowing us to understand under which circumstances livestock-based interventions could play a role in malaria control programmes.

Our model predicts that the presence of untreated livestock will have a zooprophylactic effect in two scenarios. One is when vector population density does not increase as a result of livestock introduction. The other is when although the vector population density increases, the numbers and availability of livestock to vectors are sufficiently high (such that the resulting diversion of bites from humans to livestock can counteract the increase in vector density), or the vector mortality related with host-search pre-livestock introduction was sufficient low (such that introducing livestock causes no significant decrease on the already small search mortality). Otherwise, the introduction of livestock is predicted to increase malaria transmission.

These results are in agreement with the insights from two previous zooprophylaxis models [27], [63]. Namely, Sota & Mogi [63] also identified as key determinants of the beneficial *versus* detrimental effect of untreated livestock on malaria transmission whether the vector population had reached its maximum possible density prior to livestock introduction, and whether the density and/or availability of animal hosts were sufficiently high. It is worthwhile mentioning that these features are captured by both the present model and the Sota & Mogi [63] model although the two works differ in the approach used to model the potential detrimental impact of livestock on malaria transmission. The present work explicitly models the effect of animal or human hosts' abundance and availability on vector mortality, with consequent impact on the dynamics and density of adult vectors. Instead, Sota & Mogi [63] assumed a constant vector mortality rate, and modelled the effect of hosts abundance and availability on the probability of successful blood feeding of the vector, with consequent impact on the number of eggs laid and density of adult vectors in the future generations. Aside from the work by Sota & Mogi [63], two other previous zooprophylaxis models that addressed the effect of untreated livestock on malaria transmission [27], [65], have also explicitly modelled the effect of animal or human hosts abundance and availability on vector mortality. Saul [27] also highlighted that the effect of untreated livestock on malaria greatly depended on the magnitude of the search-related vector mortality: when this is significant, increase in livestock density could lead to increased malaria transmission, which is consistent with our results.

Regarding the insecticide-treatment of livestock, when using an insecticide without diversionary properties, any given intervention effort is predicted to achieve a stronger reduction in malaria transmission in a setting with highly zoophilic vectors (exemplified by Pakistan) than with the more anthropophilic *An. arabiensis* (illustrated by Ethiopia), as expected. Yet, the same reduction in malaria prevalence could be achieved in Ethiopia, if treating a high proportion of the livestock population with a product that has stronger and/or lost lasting insecticidal effect than what was used in Pakistan. The predicted intervention effort required to achieve a given reduction in prevalence with a non-repellent insecticide, is however, surprisingly low, and most likely unrealistic. In the Pakistan trial, a 54% reduction in prevalence was obtained, following treatment of 93% of the livestock population in the trial villages (cattle, goats, and sheep) [41]. Our results suggest that, to achieve the observed reduction in prevalence with such high treatment coverage, the insecticidal effect would need to be extremely small.

When accounting for a possible repellency effect of the insecticide, the expected benefits of the intervention decrease considerably in both settings, requiring more realistic parameter values to obtain the results observed in the Pakistan trial. Repellency threshold probabilities were identified above which the intervention could become detrimental, increasing the prevalence of human infection above the pre-intervention levels. For repellency probability below those thresholds any vector diversion to humans was predicted to be overcompensated by the insecticidal (direct lethal) effect and the increased search-related mortality of the mosquitos attempting to blood feed on insecticide-treated animals. Within that range of repellency probability for which ITL is likely to still reduce malaria prevalence, a greater benefit may be observed in Pakistan or in Ethiopia, depending on the repellency and coverage levels.

The results indicate that repellency has a stronger detrimental impact on malaria (prevalence or *R*_0_) in Pakistan than in Ethiopia, and therefore, it would take a smaller level of repellency for ITL to start becoming deleterious in the Asian setting. Above the repellency threshold the intervention becomes always more detrimental in settings with higher availability of livestock to vectors, like in Pakistan and other settings with highly zoophilic vectors.

The repellency level of the insecticide applied to animals can thus have an important effect on the intervention outcome. For a given treatment coverage, the stronger and/or longer lasting the insecticidal effect, the higher is the repellency threshold above which the intervention starts becoming detrimental. Additionally, when considering doing ITL interventions with high treatment coverage of the livestock population, researchers should be aware that the higher the intervention coverage, the greater the detrimental effect from a given repellency level, and the greater the benefits from reducing repellency. A small decrease in repellency could greatly improve the intervention benefits, with the effect being greater in scenarios with more zoophilic mosquitoes. Interestingly, this is the opposite from the case of insecticide-treated nets (ITNs), where repellency could be beneficial in some circumstances. Namely, the greater the proportion of the human population covered with ITNs the greater are the expected benefits from repellency, and, conversely, the smaller the coverage the greater the likelihood that malaria vectors might be diverted from ITN-protected people to those unprotected (if the density and/or availability of animal hosts to the mosquito vector are small) [65].

In general therefore, the smaller the repellency, the greater the benefits of an ITL intervention. The benefits in settings with moderately zoophilic vectors, such as *An. arabiensis* in sub-Saharan Africa, could be further improved by artificially increasing the attractiveness of livestock to the malaria vector.

If the insecticide has diversionary properties upon the malaria vectors, the magnitude of the vector mortality related with host searching was predicted to considerable affect the model results. Namely, the smaller the vector search-related mortality pre-intervention, the stronger are the insecticide diversionary effects upon malaria prevalence or *R*_0_, be it the detrimental effect of a given repellency probability on transmission, or the reduction in transmission obtained with a given attractancy probability. Given the influential role of the vector search-related mortality upon the effects of untreated and insecticide-treated livestock on malaria transmission, obtaining field estimates for this component of vector mortality is an important challenge that future research should address.

The repellency threshold above which the intervention might become detrimental could be as low as 13% in Pakistan and 19% in Ethiopia, if assuming all livestock population is treated with an average direct insecticidal effect of 10%, under the worst case scenario of null vector search-related mortality. The smaller the treatment coverage, and/or the stronger the insecticidal effect or the search-related mortality, then the higher the repellency level at which ITL can still be safely used.

To our knowledge, this is the first modelling approach that explicitly explores the potential effects of repellency and attractancy in the context of ITL and malaria transmission. The present work is an improvement in relation to previous malaria models of the impact of applying insecticide on animals [27], on animal sheds [66], or on bednets [65]. None of the former two models [27], [66], explored a repellent or attractant effect of the insecticide, and although work by Killeen and Smith [65] has looked at repellency and livestock applied to an African setting, it did so in the context of insecticide-treated bednets and diversion of malaria vectors to humans and/or untreated cattle, without referring to insecticide-treatment of cattle.

### Considerations on modelling repellency

The present work assumes that when a mosquito tries to bite on an insecticide-treated animal and is repelled, it will be diverted to bite on another host. Nonetheless, it could be that the mosquito is not able to find a successful bloodmeal and does not feed in that night, ending up either feeding only on the following night, or dying earlier. The impact of repellency on vector mortality is captured by the model, since repellency reduces the availability of treated livestock, increasing the time required to find a bloodmeal host and consequently increasing the vector search-related mortality. The impact of repellency increasing the interval between bloodmeals is something that could be explored by extending the model to explicitly account for that possibility.

We also assume that the probability of vectors being repelled to humans after attempting to bite on livestock, depends only on the proportion of livestock population that is treated with insecticide (coverage,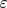), on the repellency probability of the insecticide (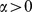), and on the relative number and availability of livestock or human hosts. Additionally, the model assumes that repellency and coverage are independent. In reality, however, the occurrence of a repellent effect can depend on additional factors such as characteristics of the: a) insecticide (chemical compound, formulation and concentration); b) intervention (concentration of the insecticide on the animal's coat, which will eventually decrease with time after application); and c) mosquito vector [73]. Also, the insecticide concentration is likely to be heterogeneous throughout the animal's surface, and the place where the mosquitoes land on the animals can therefore be determinant.

With regards to the mode of action of insecticides applied on livestock depending on the properties of the insecticide itself, some pyrethroids are more toxic to vectors than repellent (e.g. deltamethrin, used in the Pakistan ITL trial), other pyrethroids are more repellent than toxic (e.g. permethrin), and other classes of insecticides (e.g. organophosphates) are just toxic and non-repellent. Yet, even the typical toxic deltamethrin tends to be repellent at low dosages. Namely, as the applied dose of deltamethrin decays over time it goes from being toxic to non-toxic but repellent and then to just repellent.

Due to this, a big concern during the Pakistan ITL trial [41] was that mosquitoes would be repelled onto humans as the dosage of deltamethrin decayed, but it appears malaria was still controlled because the insecticide was reapplied regularly before there was too much decay. This explanation is consistent with the findings from the present work where, on one hand, when accounting for repellency the model results are more compatible with the observed Pakistan trial results, than when assuming that the insecticide had no diversion effect. On the other hand, the predictions suggest that the stronger and/or longer lasting the insecticidal effect, the highest is the repellency threshold above which the intervention is likely to become detrimental. Additionally, at the high treatment coverage applied in the trial, there is only a small difference between the repellency level with which the observed reduction in prevalence would be achieved, and the repellency threshold. This supports the hypothesis that the intervention effort applied in the Pakistan trial was sufficiently high to make the repellency effects non evident.

### Increasing livestock attractiveness to vectors

By increasing the attractiveness of insecticide treated animals to malaria vectors, it could be possible to further enhance the impact of ITL in malaria control in settings with more opportunistic vectors, such as *An. arabiensis* in Ethiopia, as shown in this work. This could eventually enable extending the geographic regions where ITL might reduce malaria burden, to include also areas with more anthropophilic vectors, such as *An. gambiae s.s.*, the most competent malaria vector in sub-Saharan Africa. Given the potential benefits this could bring, it would be worthwhile further exploring this hypothesis in future work.

In practice, however, insecticides tend to be non-attractant (i.e. neutral or repellent). Therefore, to artificially increase livestock attractiveness would require developing an insecticide that has also attractancy properties (in addition to its toxic insecticidal effect), or alternatively, treat livestock with an attractant substance on top of applying a standard insecticide. Although this may sound somewhat speculative, it is not much different from what has been successfully tested in other systems, where synthetic attractants have been applied to baits or traps to increase their attractiveness to tsetse flies [74], [75], [76], anopheline mosquitoes [58], [59], and other insects of medical and veterinary importance [77].

Regarding possible detrimental implications of artificially attracting more mosquitoes into livestock, these are likely to be minimal. Attracting a mosquito to a cow does not necessarily mean the mosquito will succeed in biting/blood feeding as it may be killed or knocked down by exposure to the insecticide before taking up blood, and that is usually the case, namely with pyrethroids. The expected reduction in mosquito survival due to increased exposure to the insecticide toxicity should actually lead to less biting. Therefore, it is unlikely there would be additional disease burden or economic costs, as long as the attractancy would be specific for malaria vectors and would not cause increased number of biting flies or other arthropods that are vectors of pathogens to livestock, and would also not cause a reduction in the animal's blood through excessive biting that could decrease milk or meat yield.

### Optimizing insecticide-treated livestock interventions

It is important to highlight that, although we explored the impact that treating livestock with insecticides could have on malaria transmission, this intervention has been traditionally used with a veterinary purpose, to control tsetse flies, ticks and other ectoparasites, and the diseases they transmit to animals, improving livestock health and productivity, such as milk and meat yield. Therefore, when evaluating the cost-effectiveness of the intervention, both the animal health benefits and the public health benefits need to be captured (‘One health’). Given the potential double side benefits of veterinary interventions like this, and given the central role of livestock in poor tropical settings, to control human disease and improve livestock health will have disproportionate economic impact that needs to be captured, as accounting for it could promote the wider implementation of the intervention. Namely, if the costs of ITL are allocated to the human health and the animal health sectors in proportion to the benefits, the intervention might be profitable and cost-effective for both sectors. Here lies a challenge to the Public Health community, which will require strengthened collaboration with the Animal Health community.

In addition to the animal health/productivity benefits, ITL uses much less insecticide than traditional malaria control methods, such as indoor spraying of houses with residual insecticide, making ITL very cheap from a human disease control perspective. In the Pakistan trial, sponging livestock with deltamethrin was shown to achieve a reduction in malaria burden similar to indoor residual spraying but with 80% less campaign costs. Furthermore, if accounting for the increase in milk production by the treated cattle, associated with clearance of tick infestations, the economic gain would be enough to cover all insecticide and labor costs [41].

The mathematical model developed here could be used to examine the economic aspects of the ‘One Health’ approach to disease control, encompassing both human and animal health benefits at a societal level. The model provides a framework for quantifying the benefits of ITL as a reduction in the human health burden (expressed as prevented DALYs, Disability Adjusted Life-Years), associated reduction in health care costs (expressed as $), as well as the improvements in animal health and productivity (expressed as $). The cost-effectiveness of ITL, accounting for both the human and veterinary benefits, could then be compared with other interventions that deliver only human health benefits, such as indoor residual spraying (IRS) and insecticide-treated bednets (ITNs), and the relative attractiveness of ITL across epidemiological settings and animal production systems examined.

The use of any animal-based intervention for malaria control will only be a component of the broader integrated malaria control approach, and will have to be deployed alongside case detection, treatment and prevention. The relative importance of animal-based interventions within the broader approach will vary between settings. Also, the adoption of any recommended intervention is intimately related to the socio-economics of the setting and it is therefore vital to understand the drivers for adoption by the target population.

In Pakistan very high treatment coverage was achieved with a free campaign and the animal owners were enthusiastic because they could see the benefits of tick elimination and improved milk and meat yield [41]. Previously to the campaign the insecticide treatment of livestock for ectoparasites was normally *ad hoc* done by householder according to perceived need, which would lead to only partial coverage at any one time. Therefore, a subsidized campaign approach is recommended, similarly to the externally funded campaigns of IRS.

Although the empirical evidence for Africa is lacking, some inferences can be made from tsetse control work. In particular, the experience from controlling human sleeping sickness in southeast Uganda by targeting the cattle reservoir of the human infective parasite shows that large scale campaigns can also reach very high (>80%) coverage levels with insecticide and trypanocidal treatment. Additionally, reducing the volume of insecticide, and so the price of ITL treatment, through restricted application protocols to target insecticide use to those areas of the cattle where tsetse or anopheline mosquitoes preferentially feed, have the potential to drive routine ITL adoption by small-holder farmers [35], [43]. To drive the private uptake of ITL usage, farmers need to see a direct benefit to their animals. Experience from the sleeping sickness work shows that for effective control through ITL it is important that the insecticide products used work against both ticks and the human disease vectors (such as synthetic pyrethroids), as tick control is often the main motivation for farmers to use ITL [34].

ITL is particularly useful for malaria control where vectors, in addition to bloodfeeding on livestock, are (or have became) exophagic (feeding outdoors, therefore escaping to ITNs exposure) and/or exophilic (resting outdoors, and thereby evading IRS).

One cannot rule out that long term and intensive use of ITL may lead to selection for anthropophily, with a consequent shift in preference from animals to humans (assuming that host preference is determined by genetic polymorphisms [78], [79], [80]). Therefore, changes in the HBI (as a proxy for host preference) should be monitored in regions where repeated campaigns are undertaken [14], [41]. Additionally, selection for anthropophily could be countered by combining ITL with indoor strategies to control anthropophilic and endophilic mosquitoes, like ITNs and IRS [19].

At the time of the field studies in the settings to which to which the ITL model was parameterized (Konso region of Ethiopia and NWFP in Pakistan) most people were not using bednets. Future work could expand the present model to investigate the use of livestock-based interventions alongside ITNs or IRS, to provide additional insights to the potential impact that combining these strategies might have on malaria transmission.

A concern inherent to any vector control intervention based on insecticides is the potential development of resistance. Namely, pyrethroid resistance is becoming increasingly wide spread across anopheline mosquitoes [81], [82] and several other arthropods that feed on livestock, such as ticks [83], [84]. It has been argued that the treatment of livestock with pyrethroids is not likely to induce stronger selection pressure for resistance in malaria vectors than insecticide-treated bednets or indoor residual spraying of houses and cattle sheds, but nevertheless, appropriate monitoring of the vector populations is required if wide scale and long term ITL interventions are implemented [14], [41], [54].

It has also been recommended that research efforts should target the identification of alternative non-pyrethroid insecticides for livestock treatment [54]. Possible candidates have recently been suggested from the avermectins class of insecticides, which have since long been used in veterinary and human medicine against several helminths and arthropod pests [85] and were latest shown to be also toxic to anopheline mosquitoes. Namely, feeding on bovine blood treated with ivermectin reduced survivorship and fecundity of *An. gambiae s.s*. and *An. arabiensis* [86], [87] and may possibly also inhibit the sporogony of *P. falciparum* as it was recently shown in treated humans [88]. Another promising avermectin is the more recent eprinomectin which has similar antihelminthic and ectoparasiticidal action as ivermectin in cattle, but with much less mammary excretion, allowing its use in pregnant and lactating animals, on the contrary of ivermectin [87]. Any of these avermectines could overcome the problems of pyrethroid resistance as well as repellency upon malaria vectors, and could be administered as part of mass livestock vaccination campaigns, simultaneously benefiting animal and human populations. Additionally, while pyrethroids can only be administered topically, both ivermectin and eprinomectine are available topically (as pour-on) and also as injectable formulation (subcutaneous administration), which could surmount the difficulty faced with pyrethroids of achieving high enough concentrations of product throughout the animal's skin. Malaria vectors would however need to bite the animal and take a bloodmeal to be exposed to the insecticide, but every biting mosquito would be exposed and die more promptly, therefore requiring a smaller dose, compared to pyrethroids. Further studies are needed to assess the effects of livestock treated with the recommended dose of ivermectin or eprinomectine upon wild populations of malaria vectors.

### Conclusions

A mathematical model was developed to predict the different effects of untreated and insecticide-treated livestock in malaria outcomes in different regions. Similarly to previous work, our model indicates that the zooprophylactic effect of untreated livestock depends on whether 1) the pre-existing malaria vector population had reached its maximum density, 2) livestock abundance and availability to the vector is sufficiently high, and 3) vector mortality related with host-search pre-livestock introduction was sufficiently low. We additional find that, as expected, the insecticide-treatment of livestock is likely to be more beneficial to humans in settings with highly zoophilic malaria vectors as in Pakistan and other areas of South Asia, than in settings with moderately zoophilic vectors, as *An. arabiensis* in sub-Saharan African. Nevertheless, the intervention could also substantially decrease malaria burden in the latter settings, under certain conditions, as illustrated here with the predictions for Ethiopia. Namely, in regions with moderately zoophilic vectors the benefits of the intervention will be maximized if 1) treating most of the livestock population with a product that has a stronger or longer lasting toxic insecticidal effect than what was used in the Pakistan trial, and that has little (ideally null) repellency effect (such as the non-pyrethroids ivermectin or eprinomectin), or 2) if the attractiveness of the treated animals to malaria vectors could be increased.

It is hoped that this work may lead to increasing awareness about the non-linear effects of livestock on malaria transmission, and to the implementation of a community-based trial of insecticide-treated livestock in an African region where *An. arabiensis* predominates, and where this strategy could potentially contribute to the integrated control of human malaria and livestock diseases.

## Supporting Information

Text S1 **Formulation of livestock effects.** S1.1. Livestock effects on Human blood index. S1.2. Livestock effects on vector mortality.
(PDF)

Text S2 **Malaria model endemic equilibrium solutions.**
(PDF)

Text S3 **Model parameterization.** S3.1. Untreated livestock model parameterization. S3.2. Insecticide-treated livestock model parameterization for Pakistan and Ethiopia.
(PDF)

Text S4 **Sensitivity analyses.** S4.1. Sensitivity analysis of the effects of untreated livestock. *Figure S1. Sensitivity analysis of the effects of livestock availability/density vs. vector search-related mortality on several malaria outcomes, at the new endemic equilibrium*. S4.2. Sensitivity analysis of the effects of insecticide-treated livestock. *Figure S2. Sensitivity analysis of the effects of repellency/attractancy vs. insecticidal probability (k) on the prevalence ratio. S4.2.1. Sensitivity analysis of the impact of vector search-related mortality upon the effects of insecticide-treated livestock. Figure S3. Sensitivity analysis of the effects of repellency/attractancy vs. vector search-mortality on the prevalence ratio. Figure S4. Sensitivity analysis of the effects of repellency/attractancy vs. vector search-mortality on the critical proportion of insecticide-treated livestock.*
(PDF)

## References

[1] Zinsstag J, Schelling E, Waltner-Toews D, Tanner M (2011) From “one medicine” to “one health” and systemic approaches to health and well-being. Prev Vet Med 101: 148–156. 20832879 10.1016/j.prevetmed.2010.07.003 PMC3145159

[2] Escalar G (1933) Applicazione sperimentale della zooprofilassi in Ardea. Rivista di Malariologia 12: 373–380.

[3] W.H.O. (1982) Manual on environmental management for mosquito control with special emphasis on mosquito vectors. Offset Publication No. 66. Geneva: World Health Organization. 283 p. 6129760

[4] Hacket LW (1937) Malaria in Europe. Oxford, UK: Oxford University Press.

[5] Brumpt E (1944) Revue critique: Zooprophylaxie du paludisme. Annales de Parasitologie Humaine et Comparée 20.

[6] Service MW (1991) Agricultural development and arthropod-borne diseases: a review. Revista de Saúde Pública 25: 165–178. 1687929 10.1590/s0034-89101991000300002

[7] W.H.O. (1991) Joint WHO/FAO/UNEP/UNCHS Panel of Experts on Environmental Management for Vector Control (PEEM): Report on the Ninth (1989) and Tenth (1990) Meetings. Geneva, Switzerland: World Health Organization. WHO–CWS/91.7 WHO–CWS/91.7. 28 p.

[8] Bettini S, Romi R (1998) [Zooprophylaxis: old and new problems]. Parassitologia 40: 423–430. 10645554

[9] Franco AIO (2010) Effects of livestock management and insecticide treatment on the transmission and control of human malaria. PhD thesis. London School of Hygiene and Tropical Medicine, University of London, United Kingdom.

[10] Charlwood JD, Dagoro H, Paru R (1985) Blood-feeding and resting behaviour in the *Anopheles punctulatus* Dönitz complex (Diptera: Culicidae) from coastal Papua New Guinea. Bulletin of Entomological Research 75: 463–475.

[11] Burkot TR, Dye C, Graves PM (1989) An analysis of some factors determining the sporozoite rates, human blood indexes, and biting rates of members of the *Anopheles punctulatus* complex in Papua New Guinea. The American Journal of Tropical Medicine and Hygiene 40: 229–234. 2929848 10.4269/ajtmh.1989.40.229

[12] van der Hoek W, Konradsen F, Dijkstra DS, Amerasinghe PH, Amerasinghe FP (1998) Risk factors for malaria: a microepidemiological study in a village in Sri Lanka. Transactions of the Royal Society of Tropical Medicine and Hygiene 92: 265–269. 9861392 10.1016/s0035-9203(98)91003-3

[13] Bouma M, Rowland M (1995) Failure of passive zooprophylaxis: cattle ownership in Pakistan is associated with a higher prevalence of malaria. Transactions of the Royal Society of Tropical Medicine and Hygiene 89: 351–353. 7570859 10.1016/0035-9203(95)90004-7

[14] Hewitt S, Kamal M, Muhammad N, Rowland M (1994) An entomological investigation of the likely impact of cattle ownership on malaria in an Afghan refugee camp in the North West Frontier Province of Pakistan. Medical and Veterinary Entomology 8: 160–164. 8025324 10.1111/j.1365-2915.1994.tb00156.x

[15] Russel PF (1934) Zooprophylaxis failure. An experiment in the Philipines. Rivista di Malariologia 13: 610–616.

[16] Schultz GW (1989) Animal influence on man-biting rates at a malarious site in Palawan, Philippines. The Southeast Asian Journal of Tropical Medicine and Public Health 20: 49–53. 2505393

[17] Ghebreyesus TA, Haile M, Witten KH, Getachew A, Yohannes M, et al (2000) Household risk factors for malaria among children in the Ethiopian highlands. Transactions of the Royal Society of Tropical Medicine and Hygiene 94: 17–21. 10748890 10.1016/s0035-9203(00)90424-3

[18] Seyoum A, Balcha F, Balkew M, Ali A, Gebre-Michael T (2002) Impact of cattle keeping on human biting rate of anopheline mosquitoes and malaria transmission around Ziway, Ethiopia. East African Medical Journal 79: 485–490. 12625690 10.4314/eamj.v79i9.9121

[19] Habtewold T (2004) Interaction between *Anopheles*, cattle and human: exploration of the effects of various cattle management practices on the behaviour and control of *Anopheles arabiensis* in Ethiopia. PhD Thesis. Greenwich: University of Greenwich, U.K. 249 p.

[20] Subramanian S, Manoharan A, Sahu S, Jambulingam P, Govardhini P, et al (1991) Living conditions and occurrence of malaria in a rural community. Indian Journal of Malariology 28: 29–37. 1915982

[21] Service MW (1993) Mosquito ecology. Field sampling methods. London: Chapman & Hall.

[22] Gillies MT, De Meillon B (1968) The Anophelinae of Africa south of the Sahara. Publications of the South African Institute for Medical Research, No. 54. South African Institute for Medical Research, Johannesburg.

[23] White BN, Magayuka SA, Boreham PFL (1972) Comparative studies on sibling species of the *Anopheles gambiae* Giles complex (Diptera Culicidae) bionomics and vectorial activity of species A and species B at Segera, Tanzania. Bulletin of Entomological Research 62: 295–317.

[24] Charlwood JD, Edoh D (1996) Polymerase chain reaction used to describe larval habitat use by *Anopheles gambiae* complex (Diptera: Culicidae) in the environs of Ifakara, Tanzania. Journal of Medical Entomology 33: 202–204. 8742521 10.1093/jmedent/33.2.202

[25] Minakawa N, Mutero CM, Githure JI, Beier JC, Yan G (1999) Spatial distribution and habitat characterization of anopheline mosquito larvae in Western Kenya. The American Journal of Tropical Medicine and Hygiene 61: 1010–1016. 10674687 10.4269/ajtmh.1999.61.1010

[26] McLaughlin RE, Focks DA (1990) Effects of cattle density on New Jersey light trap mosquito captures in the rice/cattle agroecosystem of southwestern Louisiana. Journal of the American Mosquito Control Association 6: 283–286. 1973448

[27] Saul A (2003) Zooprophylaxis or zoopotentiation: the outcome of introducing animals on vector transmission is highly dependent on the mosquito mortality while searching. Malaria Journal 2: art. no.32. 10.1186/1475-2875-2-32 PMC222927 14565850

[28] Habtewold T, Walker AR, Curtis CF, Osir EO, Thapa N (2001) The feeding behaviour and *Plasmodium* infection of *Anopheles* mosquitoes in southern Ethiopia in relation to use of insecticide-treated livestock for malaria control. Transactions of the Royal Society of Tropical Medicine and Hygiene 95: 584–586. 11816425 10.1016/s0035-9203(01)90086-0

[29] Gupta S, Swinton J, Anderson RM (1994) Theoretical studies of the effects of heterogeneity in the parasite population on the transmission dynamics of malaria. Proceedings of the Royal Society of London Series B-Biological Sciences 256: 231–238. 10.1098/rspb.1994.0075 7914705

[30] Collins WE, Jeffery GM (2003) A retrospective examination of mosquito infection on humans infected with *Plasmodium falciparum* . The American Journal of Tropical Medicine and Hygiene 68: 366–371. 12685646

[31] USDA (1976)Control of insects affecting livestock. 99 p.

[32] Thomson MC (1987) The effect on tsetse flies (*Glossina* spp.) of deltamethrin applied to cattle either as a spray or incorporated into ear-tags. Tropical Pest Management 33: 329–335.

[33] Bekele J, Asmare K, Abebe G, Ayelet G, Gelaye E (2010) Evaluation of Deltamethrin applications in the control of tsetse and trypanosomosis in the southern rift valley areas of Ethiopia. Vet Parasitol 168: 177–184. 20060647 10.1016/j.vetpar.2009.11.028

[34] Bardosh K, Waiswa C, Welburn SC (2013) Conflict of interest: use of pyrethroids and amidines against tsetse and ticks in zoonotic sleeping sickness endemic areas of Uganda. Parasit Vectors 6: 204. 23841963 10.1186/1756-3305-6-204 PMC3711891

[35] Torr SJ, Maudlin I, Vale GA (2007) Less is more: restricted application of insecticide to cattle to improve the cost and efficacy of tsetse control. Medical and Veterinary Entomology 21: 53–64. 17373947 10.1111/j.1365-2915.2006.00657.x

[36] George JE (2000) Present and future technologies for tick control. Ann N Y Acad Sci 916: 583–588. 11193677 10.1111/j.1749-6632.2000.tb05340.x

[37] Foil LD, Hogsette JA (1994) Biology and control of tabanids, stable flies and horn flies. Revue Scientifique et Technique (International Office of Epizootics) 13: 1125–1158. 7711307 10.20506/rst.13.4.821

[38] Schmidtmann ET, Lloyd JE, Bobian RJ, Kumar R, Waggoner JW, et al (2001) Suppression of mosquito (Diptera: Culicidae) and black fly (Diptera: Simuliidae) blood feeding from Hereford cattle and ponies treated with permethrin. Journal of Medical Entomology 38: 728–734. 11580047 10.1603/0022-2585-38.5.728

[39] Nasci RS, McLaughlin RE, Focks D, Billodeaux JS (1990) Effect of topically treating cattle with permethrin on blood feeding of *Psorophora columbiae* (Diptera: Culicidae) in a southwestern Louisiana rice-pasture ecosystem. Journal of Medical Entomology 27: 1031–1034. 2280387 10.1093/jmedent/27.6.1031

[40] Standfast HA, Muller MJ, Wilson DD (1984) Mortality of *Culicoides brevitarsis* (Diptera: Ceratopogonidae) fed on cattle treated with ivermectin. Journal of Economic Entomology 77: 419–421. 6547733 10.1093/jee/77.2.419

[41] Rowland M, Durrani N, Kenward M, Mohammed N, Urahman H, et al (2001) Control of malaria in Pakistan by applying deltamethrin insecticide to cattle: a community-randomised trial. Lancet 357: 1837–1841. 11410192 10.1016/S0140-6736(00)04955-2

[42] Reisen WK, Milby MM (1986) Population dynamics of some Pakistan mosquitoes: changes in adult relative abundance over time and space. Annals of Tropical Medicine and Parasitology 80: 53–68. 2873798 10.1080/00034983.1986.11811984

[43] Habtewold T, Prior A, Torr SJ, Gibson G (2004) Could insecticide-treated cattle reduce Afrotropical malaria transmission? Effects of deltamethrin-treated Zebu on *Anopheles arabiensis* behaviour and survival in Ethiopia. Medical and Veterinary Entomology 18: 408–417. 15642008 10.1111/j.0269-283X.2004.00525.x

[44] Mahande AM, Mosha FW, Mahande JM, Kweka EJ (2007) Role of cattle treated with deltamethrin in areas with a high population of *Anopheles arabiensis* in Moshi, Northern Tanzania. Malaria Journal 6: 109. 17686176 10.1186/1475-2875-6-109 PMC1971710

[45] Chareonviriyaphap T (2012) Behavioral Responses of Mosquitoes to Insecticides, Insecticides - Pest Engineering, Dr. Farzana Perveen (Ed.). InTech. Available from: http://www.intechopen.com/download/get/type/pdfs/id/28262 (Accessed 1 August 2013).

[46] Lines JD, Myamba J, Curtis CF (1987) Experimental hut trials of permethrin-impregnated mosquito nets and eave curtains against malaria vectors in Tanzania. Medical and Veterinary Entomology 1: 37–51. 2979519 10.1111/j.1365-2915.1987.tb00321.x

[47] Takken W (2002) Do insecticide-treated bednets have an effect on malaria vectors? Tropical Medicine & International Health 7: 1022–1030. 12460393 10.1046/j.1365-3156.2002.00983.x

[48] Charlwood JD, Graves PM (1987) The effect of permethrin-impregnated bednets on a population of *Anopheles farauti* in coastal Papua New Guinea. Medical and Veterinary Entomology 1: 319–327. 2979548 10.1111/j.1365-2915.1987.tb00361.x

[49] Magesa SM, Wilkes TJ, Mnzava AE, Njunwa KJ, Myamba J, et al (1991) Trial of pyrethroid impregnated bednets in an area of Tanzania holoendemic for malaria. Part 2. Effects on the malaria vector population. Acta Tropica 49: 97–108. 1680284 10.1016/0001-706x(91)90057-q

[50] Githeko AK, Adungo NI, Karanja DM, Hawley WA, Vulule JM, et al (1996) Some observations on the biting behavior of *Anopheles gambiae s.s.*, *Anopheles arabiensis*, and *Anopheles funestus* and their implications for malaria control. Experimental Parasitology 82: 306–315. 8631382 10.1006/expr.1996.0038

[51] Bøgh C, Pedersen EM, Mukoko DA, Ouma JH (1998) Permethrin-impregnated bednet effects on resting and feeding behaviour of lymphatic filariasis vector mosquitoes in Kenya. Medical and Veterinary Entomology 12: 52–59. 9513939 10.1046/j.1365-2915.1998.00091.x

[52] Kolaczinski JH, Reithinger R, Worku DT, Ocheng A, Kasimiro J, et al (2008) Risk factors of visceral leishmaniasis in East Africa: a case-control study in Pokot territory of Kenya and Uganda. International Journal of Epidemiology 37: 344–352. 18184669 10.1093/ije/dym275 PMC2637948

[53] McLaughlin RE, Focks DA, Dame DA (1989) Residual activity of permethrin on cattle as determined by mosquito bioassays. Journal of the American Mosquito Control Association 5: 60–63. 2708990

[54] Hewitt S, Rowland M (1999) Control of zoophilic malaria vectors by applying pyrethroid insecticides to cattle. Tropical Medicine & International Health 4: 481–486. 10470339 10.1046/j.1365-3156.1999.00433.x

[55] Vythilingam I, Ridhawati, Sani RA, Singh KI (1993) Residual activity of cyhalothrin 20% EC on cattle as determined by mosquito bioassays. Southeast Asian J Trop Med Public Health 24: 544–548. 7909171

[56] Elliot M (1989) The pyrethroids: early discovery, recent advances and the future. Pesticide Science 27: 337–351.

[57] W.H.O. (1990) Deltamethrin. Environmental Health Criteria 97. Geneva: World Health Organization.

[58] Okumu FO, Killeen GF, Ogoma S, Biswaro L, Smallegange RC, et al (2010) Development and field evaluation of a synthetic mosquito lure that is more attractive than humans. PLoS One 5: e8951. 20126628 10.1371/journal.pone.0008951 PMC2812511

[59] Jawara M, Awolola TS, Pinder M, Jeffries D, Smallegange RC, et al (2011) Field testing of different chemical combinations as odour baits for trapping wild mosquitoes in The Gambia. PLoS One 6: e19676. 21637337 10.1371/journal.pone.0019676 PMC3102657

[60] Ross R (1911) The Prevention of Malaria. London: Murray.

[61] Macdonald G (1952) The analysis of equilibrium in malaria. Tropical Diseases Bulletin 49: 813–829. 12995455

[62] Nåsell I (1985) Hybrid Models of Tropical Infections. In: Levin S, editor.Lecture Notes in Biomathematics. New York: Springer-Verlag. pp. 46–50.

[63] Sota T, Mogi M (1989) Effectiveness of zooprophylaxis in malaria control: a theoretical inquiry, with a model for mosquito populations with two bloodmeal hosts. Medical and Veterinary Entomology 3: 337–345. 2519684 10.1111/j.1365-2915.1989.tb00240.x

[64] Killeen GF, McKenzie FE, Foy BD, Bogh C, Beier JC (2001) The availability of potential hosts as a determinant of feeding behaviours and malaria transmission by African mosquito populations. Transactions of the Royal Society of Tropical Medicine and Hygiene 95: 469–476. 11706651 10.1016/s0035-9203(01)90005-7 PMC2483839

[65] Killeen GF, Smith TA (2007) Exploring the contributions of bed nets, cattle, insecticides and excitorepellency to malaria control: a deterministic model of mosquito host-seeking behaviour and mortality. Transactions of the Royal Society of Tropical Medicine and Hygiene 101: 867–880. 17631372 10.1016/j.trstmh.2007.04.022 PMC1949412

[66] Kawaguchi I, Sasaki A, Mogi M (2004) Combining zooprophylaxis and insecticide spraying: a malaria- control strategy limiting the development of insecticide resistance in vector mosquitoes. Proceedings of the Royal Society of London Series B-Biological Sciences 271: 301–309. 10.1098/rspb.2003.2575 PMC1691597 15058442

[67] Hurd H (2003) Manipulation of medically important insect vectors by their parasites. Annual Review of Entomology 48: 141–161. 10.1146/annurev.ento.48.091801.112722 12414739

[68] Lord CC, Woolhouse MEJ, Heesterbeek JAP (1996) Vector-borne diseases and the basic reproduction number: a case study of African horse sickness. Medical and Veterinary Entomology 10: 19–28. 8834738 10.1111/j.1365-2915.1996.tb00077.x

[69] Charlwood JD, Smith T, Kihonda J, Heiz B, Billingsley PF, et al (1995) Density independent feeding success of malaria vectors (Diptera: Culicidae) in Tanzania. Bulletin of Entomological Research 85: 29–35.

[70] Anderson RM, May RM (1991) Infectious Diseases of Humans: Dynamics and Control. Oxford: Oxford University Press.

[71] Diekmann O, Heesterbeek JA, Metz JA (1990) On the definition and the computation of the basic reproduction ratio R0 in models for infectious diseases in heterogeneous populations. Journal of Mathematical Biology 28: 365–382. 2117040 10.1007/BF00178324

[72] van den Driessche P, Watmough J (2002) Reproduction numbers and sub-threshold endemic equilibria for compartmental models of disease transmission. Mathematical Biosciences 180: 29–48. 12387915 10.1016/s0025-5564(02)00108-6

[73] IVCC. Proceedings of the Innovative Vector Control Consortium (IVCC) - Insect Repellent Workshop; 2007 22–23 January; London, UK.

[74] Rayaisse JB, Tirados I, Kaba D, Dewhirst SY, Logan JG, et al (2010) Prospects for the development of odour baits to control the tsetse flies Glossina tachinoides and G. palpalis s.l. PLoS Negl Trop Dis 4: e632. 20300513 10.1371/journal.pntd.0000632 PMC2838779

[75] Vale GA, Lovemore DF, Flint S, Cockbill GF (1988) Odour-baited targets to control tsetse flies, Glossina spp. (Diptera: Glossinidae), in Zimbabwe. Bulletin of Entomological Research 78: 31–49.

[76] Vale GA, Hall DR (1985) The role of 1-octen-3-ol, acetone and carbon dioxide in the attraction of tsetse flies, Glossina spp. (Diptera: Glossinidae), to ox odour. Bulletin of Entomological Research 75: 209–218.

[77] Chaniotis BN (1983) Improved trapping of phlebotomine sand flies (Diptera: Psychodidae) in light traps supplemented with dry ice in a neotropical rain forest. J Med Entomol 20: 222–223. 6405036 10.1093/jmedent/20.2.222

[78] Coluzzi M, Sabatini A, Petrarca V, Di Deco MA (1979) Chromosomal differentiation and adaptation to human environments in the *Anopheles gambiae* complex. Transactions of the Royal Society of Tropical Medicine and Hygiene 73: 483–497. 394408 10.1016/0035-9203(79)90036-1

[79] Donnelly MJ, Townson H (2000) Evidence for extensive genetic differentiation among populations of the malaria vector *Anopheles arabiensis* in Eastern Africa. Insect Molecular Biology 9: 357–367. 10971713 10.1046/j.1365-2583.2000.00197.x

[80] Petrarca V, Nugud AD, Ahmed MA, Haridi AM, Di Deco MA, et al (2000) Cytogenetics of the *Anopheles gambiae* complex in Sudan, with special reference to *An. arabiensi*s: relationships with East and West African populations. Medical and Veterinary Entomology 14: 149–164. 10872859 10.1046/j.1365-2915.2000.00231.x

[81] Curtis CF, Miller JE, Hodjati MH, Kolaczinski JH, Kasumba I (1998) Can anything be done to maintain the effectiveness of pyrethroid-impregnated bednets against malaria vectors? Philosophical Transactions of the Royal Society of London Series B, Biological Sciences 353: 1769–1775. 10021774 10.1098/rstb.1998.0329 PMC1692389

[82] Ranson H, N'Guessan R, Lines J, Moiroux N, Nkuni Z, et al (2011) Pyrethroid resistance in African anopheline mosquitoes: what are the implications for malaria control? Trends Parasitol 27: 91–98. 20843745 10.1016/j.pt.2010.08.004

[83] Beugnet F, Chardonnet L (1995) Tick resistance to pyrethroids in New Caledonia. Veterinary Parasitology 56: 325–338. 7754609 10.1016/0304-4017(94)00686-7

[84] Rodriguez-Vivas RI, Alonso-Díaz MA, Rodríguez-Arevalo F, Fragoso-Sanchez H, Santamaria VM, et al (2006) Prevalence and potential risk factors for organophosphate and pyrethroid resistance in *Boophilus microplus* ticks on cattle ranches from the State of Yucatan, Mexico. Veterinary Parasitology 136: 335–342. 16413971 10.1016/j.vetpar.2005.05.069

[85] Wilson ML (1993) Avermectins in arthropod vector management - prospects and pitfalls. Parasitology Today 9: 83–87. 15463716 10.1016/0169-4758(93)90210-7

[86] Fritz ML, Siegert PY, Walker ED, Bayoh MN, Vulule JR, et al (2009) Toxicity of bloodmeals from ivermectin-treated cattle to *Anopheles gambiae s.l.* . Annals of Tropical Medicine and Parasitology 103: 539–547. 19695159 10.1179/000349809X12459740922138

[87] Fritz ML, Walker ED, Miller JR (2012) Lethal and sublethal effects of avermectin/milbemycin parasiticides on the African malaria vector, Anopheles arabiensis. J Med Entomol 49: 326–331. 22493850 10.1603/me11098

[88] Kobylinski KC, Foy BD, Richardson JH (2012) Ivermectin inhibits the sporogony of Plasmodium falciparum in Anopheles gambiae. Malar J 11: 381. 23171202 10.1186/1475-2875-11-381 PMC3519548

[89] Warrel DA, Gilles HM (2002) Essential Malariology. London: Hodder Arnold. 348 p.

[90] Nedelman J (1985) Some New Thoughts About Some Old Malaria Models - Introductory Review. Mathematical Biosciences 73: 159–182.

[91] Molineaux L (1988) The epidemiology of humans malaria as an explanation of its distribution, including some implications for its control. In: Wernsdorfer WH, McGregor SI, editors. Malaria, Principles and Practice of Malariology.London: Churchill Livingstone. pp. 913–998.

[92] Mahmood F, Reisen WK (1981) Duration of the gonotrophic cycles of *Anopheles culicifacies* Giles and *An. stephensi* Liston, with observations on reproductive activity and survivorship during winter. Mosquito News 41: 22–30.

[93] Krafsur ES (1977) The bionomics and relative prevalence of *Anopheles* species with respect to the transmission of *Plasmodium* to man in western Ethiopia. Journal of Medical Entomology 14: 180–194. 606817 10.1093/jmedent/14.2.180

[94] Krafsur ES, Armstrong JC (1982) Epidemiology of *Plasmodium malariae* infection in Gambella, Ethiopia. Parassitologia 24: 105–120. 6765340

[95] Verhage DF, Telgt DS, Bousema JT, Hermsen CC, van Gemert GJ, et al (2005) Clinical outcome of experimental human malaria induced by *Plasmodium falciparum*-infected mosquitoes. The Netherlands Journal of Medicine 63: 52–58. 15768480

[96] Rickman LS, Jones TR, Long GW, Paparello S, Schneider I, et al (1990) *Plasmodium falciparum* -infected *Anopheles stephensi* inconsistently transmit malaria to humans. The American Journal of Tropical Medicine and Hygiene 43: 441–445. 2240371 10.4269/ajtmh.1990.43.441

[97] Taye A, Hadis M, Adugna N, Tilahun D, Wirtz RA (2006) Biting behavior and *Plasmodium* infection rates of *Anopheles arabiensis* from Sille, Ethiopia. Acta Tropica 97: 50–54. 16171769 10.1016/j.actatropica.2005.08.002

[98] Reisen WK, Boreham PF (1982) Estimates of malaria vectorial capacity for *Anopheles culicifacies* and *Anopheles stephensi* in rural Punjab province Pakistan. Journal of Medical Entomology 19: 98–103. 6750125 10.1093/jmedent/19.1.98

[99] Tirados I, Costantini C, Gibson G, Torr SJ (2006) Blood-feeding behaviour of the malarial mosquito *Anopheles arabiensis*: implications for vector control. Medical and Veterinary Entomology 20: 425–437. 17199754 10.1111/j.1365-2915.2006.652.x

