# Supplementary material for: Controlling Malaria Using Livestock-Based Interventions: A One Health Approach
Source: PLoS One. 2014 Jul 22;9(7):e101699. doi: 10.1371/journal.pone.0101699 (PMC4106824; doi:10.1371/journal.pone.0101699)
Supplement: Text S1 — Formulation of livestock effects. S1.1. Livestock effects on Human blood index. S1.2. Livestock effects on vector mortality. (PDF) [file pone.0101699.s001.pdf]

Text S1. Formulation of livestock effects

S1.1. Livestock effects on Human blood index

There is compelling evidence that the proportion of vectors that feed on a given host (host blood index) may vary under the influence of host and vector related factors. Accordingly, the proportion of vector bloodmeals from humans (q) was allowed to explicitly depend on the abundance and availability of alternative host types (livestock and human) to the vector population. The availability of humans can be defined as the likelihood that a vector will bite humans, if humans and livestock are equally abundant, in an area where these two host types are the only significant bloodmeal source.

In the absence of insecticide, the following relationship was used to model the proportion of vector bloodmeals on humans (after Sota and Mogi [1]):

$$q = \frac{N_h A_h}{N_h A_h + N_l A_l}$$

which can be rewritten as:

$$q = \frac{1}{1 + \frac{N_l}{N_h} \frac{A_l}{A_h}}$$

where A_h and A_l are the proportional availabilities of the human and livestock hosts, respectively, and can take any value between 0 and 1, inclusive.

Contrarily to previous models that used absolute availability values [2,3], here proportional values are used, as that overcomes the uncertainty around possible estimates of the absolute values. Therefore, throughout this work, when the term "availability" is used it will refer to "proportional availability", unless otherwise stated. A_l/A_h is the relative availability of livestock compared to humans, in an area where humans and livestock are the only significant blood sources (otherwise, for additional blood sources, the expression needs to be modified accordingly), and is equivalent to the Feeding Index defined by Kay, Boreham, and Edman [4].

The simplified expression above facilitates the process of fitting to data, because the four initial parameters are reduced to two: the ratio between livestock and human numbers (N_l/N_h), and the ratio between livestock and human availabilities (A_l/A_h). Knowing the human blood index (HBI, which corresponds to q in our model) and the (absolute or relative) abundance of hosts, the relative availability can therefore be readily estimated from the derived expression [1]:

$$\frac{A_l}{A_h} = \frac{N_h}{N_l}\left(\frac{1}{q} - 1\right).$$

In the presence of insecticide treatment, the expression for the human blood index is generalized as

$$q = \frac{1}{1 + \frac{N_l}{N_h}\frac{A_l\left(1-\varepsilon\alpha\right)}{A_h}},$$

where ε is the proportion of livestock population with insecticide at a given point in time, hereafter referred as treatment coverage, and α is the diversion probability, which is the probability that a host-seeking mosquito will be diverted away from ($\alpha > 0$, repellency) or towards ($\alpha < 0$, attractancy) an insecticide-treated animal.

The term availability includes: the accessibility of each host to the vector, the intrinsic propensity of a vector to feed upon humans *versus* animals, and to feed in the location where the host resides. In cases where cattle are kept at a considerable distance from human dwellings, this distance also changes host accessibility, consequently affecting availability. For instance, in a rice growing community where the village is surrounded by breeding sites, the effect of geographical positioning of the cattle sheds could be magnified if the cattle are at the edge of the village for example, where their encounter with malaria vectors would be significantly increased relative to situations where the cattle distribution in the villages is even, relative to human distribution. If the animals are located at the edge of the village closer to the breeding sites, their availability would increase for young susceptible vectors , but not latent vectors, which would likely be more abundant within the villages. Similarly, it would attenuate the diversion related effects of repellent insecticides if used on cattle.

S1.2. Livestock effects on vector mortality

The assumption that increases in untreated livestock relative abundance and/or availability simply decrease the HBI without affecting any other parameter would, by itself, reduce the human biting rate [HBR=(N_v/N_h)aHBI], and consequently decrease malaria transmission. However, although such zooprophylactic effect has sometimes been observed, for example in Papua New Guinea [5] and in Sri Lanka [6], the opposite has been documented in other regions, such as Ethiopia [7,8], Pakistan [9,10] and Philippines [11,12]. A possible explanation has been attributed to the impact of livestock abundance and/or availability upon vector mortality and/or density, which may vary between and even within settings.

By increasing the number of available bloodmeal hosts, such as livestock, fewer attempts may be required for vectors to obtain a successful bloodmeal. This may increase the probability of vectors having a successful bloodmeal during each gonotrophic cycle and decrease their mortality rate. The resulting increased vector survival has two epidemiological implications. Firstly, it will increase the probability of infected vectors surviving the parasite extrinsic incubation period and becoming infectious. Secondly, since

vectors can have more bloodmeals during their prolonged life, more eggs can be produced and laid, potentially generating more larvae. However, this will also lead to increased larval competition in the breeding sites [13,14,15,16,17,18], and therefore, the resulting outcome in the recruitment rate of emerging adult vectors will depend on the density-dependent constraints that may be acting.

Previous works have modelled the possible increase in malaria risk associated with the presence of untreated livestock, as being due to either an increase in vector emergence rate [1,19], or a decrease in vector mortality rate [3,20]. For the present model, the latter approach was chosen, as it enables exploring not only the resulting effect of increasing vector density, but also the effect of increasing the proportion of vectors that survive the parasite extrinsic incubation period and therefore become infectious. Accordingly, the model was expanded to incorporate: 1) variable vector mortality as a function of relative host abundance and/or availability, and 2) variable vector density as a function of the system's carrying capacity.

The model also accounts for potential repellency and attractancy effects upon vectors due to exposure to insecticide-treated livestock. Repellency is modelled assuming a worst case scenario, where vectors are diverted from ITL before sufficient exposure to a knock-down or lethal (i.e. a life expectancy changing) dose of insecticide. The model is therefore not considering situations where mosquitoes may be repelled following exposure to a dose of insecticide that has either an immediate lethal effect or a knock-down effect that induces premature death of the knocked-down mosquitoes by their predators. Repellency does however increase the vector search-related mortality, due to decreasing the availability of the insecticide-treated animals and therefore increasing the time needed to find a bloodmeal host. Conversely, attractancy decreases the search-related mortality but increases vector mortality due to the direct lethal effect of insecticide applied on livestock.

References

1. Sota T, Mogi M (1989) Effectiveness of zooprophylaxis in malaria control: a theoretical inquiry, with a model for mosquito populations with two bloodmeal hosts. Medical and Veterinary Entomology 3: 337-345.
2. Killeen GF, McKenzie FE, Foy BD, Bogh C, Beier JC (2001) The availability of potential hosts as a determinant of feeding behaviours and malaria transmission by African mosquito populations. Transactions of the Royal Society of Tropical Medicine and Hygiene 95: 469-476.
3. Saul A (2003) Zooprophylaxis or zoopotentiation: the outcome of introducing animals on vector transmission is highly dependent on the mosquito mortality while searching. Malaria Journal 2: art. no. 32.
4. Kay BH, Boreham PFL, Edman JD (1979) Application of the "feeding index" concept to studies of mosquito host-feeding patterns [to understand the epidemiology of diseases transmitted by arthropods]. Mosquito News 39: 68-72.

5. Charlwood JD, Dagoro H, Paru R (1985) Blood-feeding and resting behaviour in the *Anopheles punctulatus* Dönitz complex (Diptera: Culicidae) from coastal Papua New Guinea. Bulletin of Entomological Research 75: 463-475.

6. van der Hoek W, Konradsen F, Dijkstra DS, Amerasinghe PH, Amerasinghe FP (1998) Risk factors for malaria: a microepidemiological study in a village in Sri Lanka. Transactions of the Royal Society of Tropical Medicine and Hygiene 92: 265-269.

7. Ghebreyesus TA, Haile M, Witten KH, Getachew A, Yohannes M, et al. (2000) Household risk factors for malaria among children in the Ethiopian highlands. Transactions of the Royal Society of Tropical Medicine and Hygiene 94: 17-21.

8. Seyoum A, Balcha F, Balkew M, Ali A, Gebre-Michael T (2002) Impact of cattle keeping on human biting rate of anopheline mosquitoes and malaria transmission around Ziway, Ethiopia. East African Medical Journal 79: 485-490.

9. Bouma M, Rowland M (1995) Failure of passive zooprophylaxis: cattle ownership in Pakistan is associated with a higher prevalence of malaria. Transactions of the Royal Society of Tropical Medicine and Hygiene 89: 351-353.

10. Hewitt S, Kamal M, Muhammad N, Rowland M (1994) An entomological investigation of the likely impact of cattle ownership on malaria in an Afghan refugee camp in the North West Frontier Province of Pakistan. Medical and Veterinary Entomology 8: 160-164.

11. Russel PF (1934) Zooprophylaxis failure. An experiment in the Philipines. Rivista di Malariologia 13: 610-616.

12. Schultz GW (1989) Animal influence on man-biting rates at a malarious site in Palawan, Philippines. The Southeast Asian Journal of Tropical Medicine and Public Health 20: 49-53.

13. Schneider P, Takken W, McCall PJ (2000) Interspecific competition between sibling species larvae of *Anopheles arabiensis* and *An. gambiae*. Medical and Veterinary Entomology 14: 165-170.

14. Reisen WK (1975) Intraspecific competition in *Anopheles stephensi* Liston. Mosquito News 35: 473-482.

15. Koenraadt CJM, Takken W (2003) Cannibalism and predation among larvae of the *Anopheles gambiae* complex. Medical and Veterinary Entomology 17: 61-66.

16. Reisen WK, Emory RW (1977) The effects of larval intraspecific competition on imaginal densities in *Anopheles stephensi* (Diptera: Culicidae): A laboratory evaluation. Canadian Entomology 109: 1481-1484.

17. Reisen WK, Emory RW (1977) Intraspecific competition in *Anopheles stephensi* (Diptera: Culicidae). II. The effects of more crowded densities and the addition of antibiotics. Canadian Entomology 109: 1475-1480.

18. Gimnig JE, Ombok M, Otieno S, Kaufman MG, Vulule JM, et al. (2002) Density-dependent development of *Anopheles gambiae* (Diptera: Culicidae) larvae in artificial habitats. Journal of Medical Entomology 39: 162-172.

19. Kawaguchi I, Sasaki A, Mogi M (2004) Combining zooprophylaxis and insecticide spraying: a malaria- control strategy limiting the development of insecticide resistance in vector mosquitoes. Proceedings of the Royal Society of London Series B-Biological Sciences 271: 301-309.

20. Killeen GF, Smith TA (2007) Exploring the contributions of bed nets, cattle, insecticides and excitorepellency to malaria control: a deterministic model of mosquito host-seeking behaviour and mortality. Transactions of the Royal Society of Tropical Medicine and Hygiene 101: 867-880.
