# Supplementary material for: Controlling Malaria Using Livestock-Based Interventions: A One Health Approach
Source: PLoS One. 2014 Jul 22;9(7):e101699. doi: 10.1371/journal.pone.0101699 (PMC4106824; doi:10.1371/journal.pone.0101699)
Supplement: Text S2 — Malaria model endemic equilibrium solutions. (PDF) [file pone.0101699.s002.pdf]

Text S2. Malaria model endemic equilibrium solutions

The endemic equilibrium solutions were derived by solving the model system equations (1)-(2) in Maple 7(© Waterloo Maple Inc. 2001). The human system was simplified by assuming that $S_h=N_h-I_h$, which enabled to omit the differential equation for S_h and solve the remaining model equations.

The endemic equilibrium is given by

$(S_h^*, I_h^*, S_v^*, L_v^*, I_v^*)$,

where:

$$S_h^* = N_h - I_h$$

$$I_h^* = \frac{N_h(-N_h\omega r\mu^2 - N_h\mu^3 r + a^2q^2b\omega\rho N_v c)}{(N_h\omega r\mu + N_h\mu^2 r + aqb\omega\rho N_v)aqc}$$

$$S_v^* = \frac{N_h\omega r\mu + N_h\mu^2 r + aqb\omega\rho N_v}{aqb\omega(aqc+\mu)}$$

$$L_v^* = \frac{-N_h\omega r\mu^2 - N_h\mu^3 r + a^2q^2b\omega\rho N_v c}{aqb(aqc+\mu)(\omega+\mu)\omega}$$

$$I_v^* = \frac{-N_h\omega r\mu^2 - N_h\mu^3 r + a^2q^2b\omega\rho N_v c}{aqb(aqc+\mu)(\omega+\mu)\mu},$$

and

$$q = \frac{1}{1+\frac{N_l}{N_h}\frac{A_l(1-\varepsilon\alpha)}{A_h}}$$

$$\mu = \mu_m + \left(\frac{1}{(N_hA_h + (1-\varepsilon\alpha)N_lA_l)j}\right)a + \left(\frac{\varepsilon(1-\alpha)N_lA_l}{N_hA_h + N_lA_l}k\right)a\ .$$

The incorporation of explicit density dependence on vector recruitment requires replacing N_v by its equilibrium level, N_v^*. In the model where vector density is kept constant $N_v^* = N_v(0)$, while in the model with variable vector density $N_v^* = \frac{(\rho_0 - \mu)}{\rho_0}K$.
