# Supplementary material for: Controlling Malaria Using Livestock-Based Interventions: A One Health Approach
Source: PLoS One. 2014 Jul 22;9(7):e101699. doi: 10.1371/journal.pone.0101699 (PMC4106824; doi:10.1371/journal.pone.0101699)
Supplement: Text S3 — Model parameterization. S3.1. Untreated livestock model parameterization. S3.2. Insecticide-treated livestock model parameterization for Pakistan and Ethiopia. (PDF) [file pone.0101699.s003.pdf]

Text S3. Model parameterization

S3.1. Untreated livestock model parameterization

All simulations for untreated livestock used the parameter values listed in Table 1, unless otherwise stated. These values were chosen such that, in the endemic equilibrium, when livestock are present with the same density and availability as humans, there is still malaria infection (I_h^*=1.2% and I_v^*=0.6%)[1]. The initial value of the vector population density prior to increase in numbers of livestock is denoted by $N_v(0)$.

Density of livestock and human hosts (N_l, N_h)

The ratio of livestock to human density was varied by changing the number of livestock while fixing the number of humans equal to 100. Therefore, the increase in ratio of livestock to human density corresponds always to an increase in the absolute number of livestock.

Availability of livestock and human hosts (A_l, A_h)

To transform the proportional availabilities into the *absolute availability* values required for estimating μ_s, a *scaling factor* (*j*) was used. An analytical expression was devised to derive *j* for any setting with known abundance and proportional availability of humans (N_h and A_h, respectively), vector biting rate (a), vector mortality rate in the absence of livestock (μ_h) and a hypothetical or estimated μ_s, as follows.

Knowing that

$$\mu_h = \mu_m + \left(\frac{a}{(N_h A_h) j} \right) ,$$

where the last term is the vector search-related mortality (μ_s) pre-livestock introduction, by solving for *j* it is possible to derive the following expression:

[1] In order to obtain persisting levels of infection when livestock are present with the same density and availability as humans, that means that, when livestock are absent the prevalence of infection in humans and vectors is considerably high: I_h^*=39.4% and I_v^*=15.5%. Although this prevalence of infection in vectors is higher than the values found in most areas of the world (usually <1%), it lies within observed ranges for the African species in the *An. gambiae* complex and *An. funestus* (normally from 1 to 5% but may approach 10-30% during certain times of the year) [1,2,3].

$$j = \frac{a}{(N_h A_h)(\mu_h - \mu_m)}.$$

Letting $\mu_m = x\mu_h$, the expression for j becomes equivalent to:

$$j = \frac{a}{(N_h A_h)(\mu_h (1-x))}.$$

The model for untreated livestock used the latter expression to derive j, where x is the proportion of the vector mortality in the absence of available livestock that is unrelated with searching for a bloodmeal host (and, conversely, 1-x, also designated by ψ, is the proportion of vector mortality in the absence of available livestock related with host search).

The values of j were chosen to obtain μ_h=0.1/day (which is illustrative and within the limits of recorded field values – vector life expectancy of 10 days), in a village with 100 persons and where the vector feeds once every two days (j=0.1105, 0.2 and 1 for A_h=0.9, 0.5 and 0.1, respectively – which corresponds to A_l=0.1, 0.5 and 0.9, respectively, in the baseline scenario of $x=\psi$ =0.5).

S3.2. Insecticide-treated livestock model parameterization for Pakistan and Ethiopia

The parameter values used to explore the effects of insecticide-treated livestock are listed in Table 2. Most values were either extracted or derived from empirical data from the index studies in the NWFP of Pakistan (ITL trial by Rowland et al. [4]) and in the Konso district of South-west Ethiopia (field study by Franco [5]), or from previous studies within or near the area of the index studies. When data for a parameter were available from more than one published study, the simulations used the estimates from the studies that were most recent and/or conducted in areas within or closest to the areas of the index studies.

Rational for selection of *Plasmodium falciparum*

We model infection by *Plasmodium falciparum* because it is the most serious form of malaria infection and has no relapses, unless due to treatment failure, while with *P. vivax* the more frequent relapses may obscure the effects of the intervention, and other species are less prevalent. Additionally, in the Ethiopian setting most cases were due to *P. falciparum* [5]. Although in the Pakistan trial setting, *P. vivax* infections were more frequent [4], for comparison purposes, only the trial data for *P. falciparum* were used in the simulations.

Rational for selection of the species of malaria vectors

Pakistan: In the area of the Pakistan ITL trial the main malaria vectors were *An. culicifacies* and *An. stephensi* [6]. The simulations considered only *An. culicifacies* because this species is more anthropophilic, has a longer life expectancy, and has been implied as the most important vector species in studies done in Punjab Province, near the ITL trial study area [7]. We are therefore simulating a worse-case scenario regarding the vector species.

Ethiopia: The model was fitted to *An. arabiensis*, as this is known to be the most important malaria vector in the study area, as well in the rest of the country [8,9,10].

Malaria prevalence in the study areas

Pakistan: The prevalence of *P. falciparum* infection in people in non-intervention villages during the ITL trial, ranged from 0.3% to 7.8% (M. Rowland, unpublished data). The simulations were conducted using baseline infection prevalence in people of 6%. This higher end range was chosen for three reasons: (1) it fell within the observed prevalence range; (2) however, as the villages chosen for the ITL trial had been subject to recurrent

indoor spraying campaigns [4][1], the observed prevalence range was on average lower than expected in the majority of communities not experiencing regular vector control interventions and so, to understand the potential effects of the ITL intervention it is important to examine the higher prevalence settings as that enables quantifying the full potential public health benefits of targeting livestock; (3) from a theoretical perspective, the chosen starting condition allows examining a greater range of prevalence dynamics than starting at the lower end of the observed prevalence range.

Ethiopia: To the best of our knowledge at the date of this study, no data existed on the precise prevalence of *Plasmodium spp.* infection in the Ethiopian setting. However, since the figures were likely to be higher in Ethiopia than in Pakistan, simulations were done with a conservative baseline prevalence of infection in people of 10%. The corresponding predicted prevalence of sporozoite infection in mosquitoes was 0.38 %. This is consistent with field estimates in a village (Fuchucha) of the Ethiopian setting, where Tirados et al. [10] found that the *P. falciparum* sporozoite prevalence in samples of *An. arabiensis* was 0.38% and 0.18%, for mosquitoes attracted only to human or only to cattle baits, respectively (the overall *P. falciparum* sporozoite prevalence was 0.33%).[2]

Human recovery rate from infection (*r*)

The human recovery rate from infection is given by 1/(average duration of infection in humans). The average duration of infection was based on the time elapsed since start of malaria symptoms until arriving at a health facility to receive treatment. During the Pakistan ITL trial this would take around two weeks (M. Rowland, unpublished data), and was inflated to 21 days to account for situations where it may have taken longer until receiving treatment. The same baseline value was assumed for Ethiopia, to facilitate the comparison of the predicted results with the Pakistan setting. This corresponds to a recovery rate of 0.05/day.

Rate at which infected mosquitoes become infectious (ω)

The rate at which infected mosquitoes become infectious is given by 1/(average duration of latent period in vector), which was estimated using Moshkovsky's formula [11] for *P. falciparum*: latent period = 111/(T-16), $18<T<30$, where T is the mean temperature in Celsius. The temperature data used were recorded near the Pakistan ITL trial setting at the Peshwara Meteorological Station, and in the center of the Ethiopian setting at the Karat

[1] The only villages included in the study were those whose Annual Parasite Index (incidence per year) had decreased below the threshold for indoor spraying, by permission of the United Nations High Commissioner for Refugees (UNHCR). In these villages malaria incidence had fallen to its lowest level for 5 years due to recurrent indoor spraying campaigns.

[2] Samples were taken in 2003 from the June and July catches, which corresponds to the second half of the peak in density, when mosquitoes might be expected to be older generally and therefore more likely to be infected.

Meteorological Station, and were obtained by personal communication with the National Meteorological Station in each country [5].

Pakistan: The average of the monthly mean temperatures recorded in Peshwara during the three years of the ITL was 22.3 ºC, which predicts a latency period of 17.5 days (range: 6.6 days in June to 18.9 days in April), giving ω=0.057/day.

Ethiopia: The average of the monthly mean temperatures recorded in Karat during the year of the index study was 23.1 ºC, corresponding to a latency period of 15.6 days (range: 11.9 days in March to 19.0 days in June), giving ω=0.064/day.

Infection probability of humans (*b*)

The value of the infection probability of humans was based on findings from two separate studies where 5 out of 10 volunteers have developed parasitaemia after being bitten by one or two *An. stephensi* infected with *P. falciparum* [12,13]. No data were found for *An. culicifacies,* and therefore the *An. stephensi* data were assumed. Similar patterns of sporozoite transmission between *An. stephensi* and *An. gambiae* have been proposed [14]. Accordingly, *b* was set to 0.5 for both the Pakistan and Ethiopian simulations.

Infection probability of vectors (*c*) and relative density of vectors to humans (N_v/N_h)

As in previous vector-borne disease models [e.g. 15,16], the values for the infection probability of vectors (*c*), and the relative density of vectors to humans (N_v/N_h) were chosen to produce malaria prevalence levels similar to the observed in the study areas, giving c=0.95, N_v/N_h=50 in Pakistan, and c=0.07 and N_v/N_h=15 for Ethiopia. Also, it was assumed that the density of vectors pre-intervention was at equilibrium.

Vector biting rate (*a*)

The vector biting rate on any host (*a*) was estimated assuming that the interval between bloodmeals on any host corresponds to the duration of the vector gonotrophic cycle (*g*).

Pakistan: The mean duration of the gonotrophic cycle for *An. culicifacies* was based on a study conducted in the Pakistan Punjab Province near the ITL area, that determined that after the first gonotrophic cycle which may take 4 days, the other cycles took 2 days in Summer (from Aug -Oct), and 3 days in Winter (Nov-Dec) [17].

Ethiopia: Findings from a study done in Gambella, an Administrative Region west bordering the Konso District, suggested a 2-days interval between feeding and oviposition for *An. arabiensis* [18]. Studies in North-Eastern Tanzania demonstrated a 3-days interval

for the closely-related *An. gambiae s.s.* [19] and *An. funestus* [20]. In a later study, also in Gambella, hypothetical sporozoite prevalences have been estimated using a 2 to 3-days interval [21].

Accordingly, for both Pakistan and Ethiopia simulations, g was set to 2.5 days, giving a= 0.4/day.

Vector natural mortality rate (μ_0)

The vector natural mortality rate (μ_0) is given by 1/(average vector life expectancy), which was derived with the standard formula [22,23]:

average life expectancy = $-1/\ln(p) = -1/\ln(Pr^{1/g})$

where p is the probability of daily survival, Pr is the proportion of parous female mosquitoes, and g is the mean duration of the gonotrophic cycle. It is valid to use the proportion parous to determine vector survival providing the following are observed: the population has reached a stationary age distribution; the survival rate is the same for all age groups; different age groups are sampled with similar efficiency; and the gonotrophic cycle duration is known and is constant [24]. Although in reality these conditions are rarely met, they are assumed to have been the case, as done in previous zooprophylaxis models.

Pakistan: The proportion parous of *An. culicifacies* was 0.58, as determined by Rowland et al. [4, complemented with unpublished data], from indoors resting mosquitoes collected in one of the untreated villages, from July to December in the second year of the ITL trial[1]. The estimated vector life expectancy was therefore 4.63 days, resulting in μ_0 =0.22/day.

Ethiopia: The value of proportion parous used was 0.732, which is the average of the proportion parous estimated from October 2001 to August 2002, by Taye et al [25], from indoors and outdoor human landing collections of *An. arabiensis* female mosquitoes in Sille town, near the Konso District. The corresponding life expectancy was 8.01 days, giving μ_0 =0.12/day.

In both the Pakistan and Ethiopian settings the proportion parous was determined by the authors (Rowland et al. [4], and Taye et al. [25], respectively) using the ovarioles tracheolar method of Deltinova [26].

[1] Only the second year of the trial was considered, because in this year a higher number of mosquitoes was dissected than in the first year of the trial when parous rates were also determined.

Density of livestock and human hosts (N_l, N_h)

Since for the purpose of the simulations it is sufficient to use relative density of hosts, a density of 100 persons/hectare was considered, for illustrative purposes. The relative density of livestock to humans was 0.14 in the Pakistan [4] and 1.13 in the Ethiopian [5] index study settings.

The present work accounts not only for cattle but also for other types of livestock, namely sheep and goats, as in the Pakistan and Ethiopian settings these were the alternative sources of bloodmeal for malaria vectors and were also treated with insecticide. For simplicity, it was assumed that one head of cattle was equivalent to one sheep or goat.

The relative availability of each of these animal types may however be different, not only because different animal types may be kept in different locations (at different distances from people's sleeping room and from vector breeding sites), but also because the vector's feeding preference and even feeding success may differ between animal types. For example, a study in Pakistan has found that, despite malaria vectors preferred to feed on goats than on cattle, feeding success was smaller on the former than on the latter hosts [27]. Such differences could be accounted by explicitly decomposing the availability term to equal the sum of the availabilities of each of these animal kinds, weighted averaged by their proportional abundance. The proportional availability of each animal type could be estimated if knowing the proportion of blood-meals taken upon each animal type. Alternatively, one could roughly assume that one head of cattle is equivalent to two sheep/goats, as done in experimental studies [28]. In the context of the present model, changing the assumptions about the relative contribution of each animal type to the overall abundance of livestock hosts would change: the relative density of animals per human, the estimated host availabilities, the *HBI*, and the availability scaling factor *j*, consequently affecting also the predicted vector mortality due to host search.

For the Pakistan simulations the implications would be minimal because most livestock were cattle. For Ethiopia, however, the impact would be greater, as the density of sheep/goats was more than twice than cattle. The implications of these different assumptions are described elsewhere [5], where comparisons were made of the above mentioned parameters when considering that (i) one head of cattle is equivalent to one sheep/goat, like done in the present manuscript, or (ii) one head of cattle is equivalent to two sheep/goats, or (iii) when considering only cattle. Basically, from i) to ii) and from ii) to iii), the values of the relative density of livestock/human would decrease, while the relative availability of livestock per humans, HBI, and *j* would increase.

Availability of livestock and human hosts (A_l, A_h)

i) The *relative availability of livestock to humans* (A_l / A_h) in a given setting can be estimated from the relative abundance of hosts and the HBI, using the formula based in Sota & Mogi [29]:

$$\frac{A_l}{A_h} = \frac{N_h}{N_l}\left(\frac{1}{HBI} - 1\right)$$

Since there were no data on the HBI for the Pakistan ITL trial area, nor for the whole of the Ethiopian study area, the A_l/A_h was estimated using the relative abundance of hosts and HBI from previous studies conducted in the Pakistan Punjab Province near the ITL area (study in 1978 by Reisen and Boreham [6]), and in the Ethiopian Fuchucha village, within the index study area (study in 2003 by Tirados et al. [10]).

ii) Having the value for A_l/A_h allows also to easily derive the *proportional availability* of each type of host (A_l and A_h) as follows.
Let $\theta_{Al} = A_l / A_h$,
by replacing A_h=1-A_l in the above equation for θ_{Al}, and doing algebraic manipulation, then

$$A_l = \frac{\theta_{Al}}{1+\theta_{Al}},$$

resulting in A_l = 0.982 for *An. culicifacies* in Pakistan and 0.484 for *An. arabiensis* Ethiopia.

iii) To transform the proportional availabilities into the *absolute availability* values required for estimating μ_s, a scaling factor (*j*) was used, that was derived in a similar way to that for the untreated livestock model. Here, an analytical expression was derived for *j* that allows this parameter to be estimated for any setting with known abundance of hosts (N_l and N_h), HBI (which enables estimating the proportional availability of hosts, A_l and A_h), vector biting rate (*a*), and proportion parous (which enables estimating the natural vector mortality rate, μ_0), and a hypothetical or estimated μ_m.

Knowing that

$$\mu_0 = \mu_m + \left(\frac{a}{(N_h A_h + N_l A_l)j}\right),$$

where the last term is the background vector search-related mortality (i.e. μ_s when no livestock are treated with insecticide), by solving for *j* it is possible to derive the following expression:

$$j = \frac{a}{(N_h A_h + N_l A_l)(\mu_0 - \mu_m)}.$$

Letting $\mu_m = x'\mu_0$ the expression for *j* becomes equivalent to:

$$j = \frac{a}{(N_h A_h + N_l A_l)(\mu_0(1-x'))}.$$

The model used the latter expression for *j*, where x' is the proportion of the vector natural mortality (μ_0) that is unrelated with searching for a bloodmeal host (and, conversely, $1-x'$, also designated by ψ', is the proportion of the vector natural mortality related with host search).

Pakistan: The estimated A_l/A_h was 53.24 for *An. culicifacies* (Table S1).

Ethiopia: From the estimated A_l/A_h of 0.94 for *An. arabiensis* it is noticeable that, despite in Fuchucha livestock were ~50% more abundant than humans, the proportional availability of livestock (A_l=0.48) was slightly lower than that of humans (A_h=0.52) (Table S2), which is consistent with the anthropophilic behaviour that has been demonstrated for *An. arabiensis* in the study area [10].

Vector minimum mortality rate (μ_m) and search-related mortality (μ_s)

The vector minimum mortality rate (μ_m, when there are no hazards due to searching for a bloodmeal host) was conservatively assumed to be half of the average vector natural mortality (μ_0) observed in Pakistan and also in Ethiopia (i.e. $x'=\psi'=0.5$), which results in $\mu_m=\mu_s$ pre-intervention=0.11/day for *An. culicifacies* and 0.06 for *An. arabiensis* (corresponding to a maximum longevity of 9.26 and 16.02 days, respectively).

A sensitivity analysis was done to access the impact that alternative relative magnitudes of μ_m and μ_s could have on the predicted outcomes, by exploring a conservative wider range than the one that is likely to occur in many natural settings. For that purpose, parameter x' was varied from 0.10 to 1 (i.e. ψ' varied from 0.90 to 0), which corresponds to μ_m varying from 0.022 to 0.220 /day for *An. culicifacies*, and 0.012 to 0.120/day for *An. arabiensis* (background μ_s varying from 0.198 to 0, and 0.108 to 0, respectively), using the values for μ_0, *a*, N_h, N_l, A_h and A_l in Table 2.

Vector human blood index (HBI)

The HBI for the vector in each setting was estimated using the A_l/A_h derived above from data on previous studies [6,10] and the N_l/N_h from the index studies [4,5], using the standard formula:

$$HBI = \frac{1}{1+\frac{N_l}{N_h}\frac{A_l}{A_h}}$$

Pakistan: The predicted HBI for *An. culicifacies* in the ITL trial setting (11.8%) was more than twice the HBI that had been estimated near the trial study area in the past (4.8% [6]) (Table S1). Such difference may be due to the higher relative density of humans:livestock during the ITL trial setting, which was also more than the double than during the previous study by Reisen and Boreham [6].

Ethiopia: The predicted overall HBI for *An. arabiensis* in the study area (49%; 95% CI= 39%-64%; Table S2) is concordant with previous estimates of HBI for Fuchucha (~49% by Habtewold [9]) and for other areas in Ethiopia (~40% by Hadis et al. [30]).

Table S1. Estimating the relative and proportional availabilities of livestock and human hosts, vector HBI and natural mortality rate, and availability scaling factor, for *An. culicifacies* in Pakistan.

Parameter	Previous studies nearby		Index study setting
N_l/N_h	**0.37**[a]		**0.14**[c]
HBI	**0.048**[a]		0.118
A_l/A_h	53.24	→	53.24
A_l	0.982	→	0.982
A_h	0.018	→	0.018
g	**2.5**[b]	→	**2.5**[b]
Pr	-		**0.583**[c]
μ_0	-		0.22
ψ'	-		0.5
j	-		0.238

Figures in bold are from observed data: [a] Reisen & Boreham [6]; [b] Mahmood & Reisen [17]; [c] Rowland et al. [4]. The remaining values were derived as illustrated with the slashed arrows and explained in the text, except ψ' which was hypothetically set to 0.5. The full arrows denote parameters values that were assumed to be the same in the index study (ITL trial area) as in the previous studies nearly (Punjab Province).

Table S2. Estimating the relative and proportional availabilities of livestock and human hosts, vector HBI and natural mortality rate, and availability scaling factor, for *An. arabiensis* in Ethiopia.

Parameter	Previous studies nearby/within		Index study setting
N_l/N_h	**1.49**[a]		**1.13 (0.61-1.64)**[d]
HBI	**0.417**[a]		0.485 (0.394-0.636)
A_l/A_h	0.938	→	0.938
A_l	0.484	→	0.484
A_h	0.516	→	0.516
g	**2.5**[b]	→	**2.5**[b]
Pr	**0.732**[c]	→	**0.732**[c]
μ_0	0.12	→	0.12
ψ'	-		0.5
j	-		0.060 (0.049-0.079)

Figures in bold are from observed data: [a] Tirados et al. [10]; [b] Krafsur & Armstrong [21]; Krafsur [18]; [c] Taye et al. [25]; [d] Franco [5]. The remaining values were derived as illustrated with the slashed arrows and explained in the text, except ψ' which was hypothetically set to 0.5. The full arrows denote parameters values that were assumed to be the same in the index study area (Konso district) as in previous studies in within or nearby areas. N_l/N_h is the mean ratio of the number of animals per person calculated from the number of animals/person in each individual household. In the study by Tirados et al. [10] (a) all the households of the Fuchucha village were sampled, while in the index study (d), that includes Fuchucha plus 7 other villages (Dokatu, Duraite, Nalaya Segen, Sorobo, Gamole, Buso, Mechelo, and Baide), only some houses were sampled in each village, and therefore the sampling weighted mean N_l/N_h is presented. The weight of each village was calculated dividing the total number of households in a village by the number of households interviewed in that village. Inside brackets are 95% CI.

Insecticidal probability (*k*)

Our model assumes the insecticide effects (insecticidal and diversionary probabilities) are constant, therefore reflecting average values of what would be observed throughout the year. The insecticide direct lethal effect upon vectors exposed to treated livestock (denoted as insecticidal probability, parameter *k*) was estimated based on whole-animal bioassays of deltamethrin applied to cattle in Pakistan, where the proportion of female anopheline mosquitoes dead or unfed was ~85% on the day after treatment, decreasing to 50% after two weeks [31]. To account for the decay of insecticide residual activity, the *k* value was estimated as the weighted arithmetic average ($\bar{k}$) of the insecticide lethal probability observed in the bioassay, calculated over the eight month period of active transmission of *P. falciparum* in the Pakistan trial villages (July to February, 245 days [5]). We considered three rounds of insecticide treatment of livestock during that period, with 6 weeks interval between rounds, like in the trial [4]. Namely, $\bar{k}$ was calculated as:

$$\bar{k} = \sum_{k} f(k).k ,$$

where $f(k)$ is the frequency of each *k* value during the active transmission period (i.e. $f(k) =$ number of days when a given *k* value was observed divided by 245 days). Unfortunately, the available bioassay data for the proportion of anopheline mosquitoes dead or unfed could not be dissociated, and therefore it is not possible to know the actual proportion dead/knockdown and the proportion unfed. Given this, the true observed insecticidal probability is likely to have been smaller than our estimated $\bar{k}$ of 0.12. Accordingly, throughout the manuscript we refer to a slightly smaller estimated *k* of 0.10.

References

1. Beier JC (1998) Malaria parasite development in mosquitoes. Annual Review of Entomology 43: 519-543.
2. Beier JC, Oster CN, Onyango FK, Bales JD, Sherwood JA, et al. (1994) *Plasmodium falciparum* incidence relative to entomologic inoculation rates at a site proposed for testing malaria vaccines in western Kenya. The American Journal of Tropical Medicine and Hygiene 50: 529-536.
3. Beier JC, Perkins PV, Koros JK, Onyango FK, Gargan TP, et al. (1990) Malaria sporozoite detection by dissection and ELISA to assess infectivity of afrotropical *Anopheles* (Diptera: Culicidae). Journal of Medical Entomology 27: 377-384.
4. Rowland M, Durrani N, Kenward M, Mohammed N, Urahman H, et al. (2001) Control of malaria in Pakistan by applying deltamethrin insecticide to cattle: a community-randomised trial. Lancet 357: 1837-1841.
5. Franco AIO (2010) Effects of livestock management and insecticide treatment on the transmission and control of human malaria. PhD thesis. London School of Hygiene and Tropical Medicine, University of London, United Kingdom.
6. Reisen WK, Boreham PF (1982) Estimates of malaria vectorial capacity for *Anopheles culicifacies* and *Anopheles stephensi* in rural Punjab province Pakistan. Journal of Medical Entomology 19: 98-103.

7. Mahmood F, Sakai RK, Akhtar K (1984) Vector incrimination studies and observations on species A and B of the taxon *Anopheles culicifacies* in Pakistan. Transactions of the Royal Society of Tropical Medicine and Hygiene 78: 607-616.
8. Habtewold T, Walker AR, Curtis CF, Osir EO, Thapa N (2001) The feeding behaviour and *Plasmodium* infection of *Anopheles* mosquitoes in southern Ethiopia in relation to use of insecticide-treated livestock for malaria control. Transactions of the Royal Society of Tropical Medicine and Hygiene 95: 584-586.
9. Habtewold T (1999) Prospects of using insecticide treated cattle for tsetse-anophelines integrated control. MSc Dissertation. Center for Tropical Veterinary Medicine: University of Edinburgh, U.K. 149 p.
10. Tirados I, Costantini C, Gibson G, Torr SJ (2006) Blood-feeding behaviour of the malarial mosquito *Anopheles arabiensis*: implications for vector control. Medical and Veterinary Entomology 20: 425-437.
11. Molineaux L (1988) The epidemiology of humans malaria as an explanation of its distribution, including some implications for its control. In: Wernsdorfer WH, McGregor SI, editors. Malaria, Principles and Practice of Malariology. London: Churchill Livingstone. pp. 913-998.
12. Rickman LS, Jones TR, Long GW, Paparello S, Schneider I, et al. (1990) *Plasmodium falciparum* - infected *Anopheles stephensi* inconsistently transmit malaria to humans. The American Journal of Tropical Medicine and Hygiene 43: 441-445.
13. Verhage DF, Telgt DS, Bousema JT, Hermsen CC, van Gemert GJ, et al. (2005) Clinical outcome of experimental human malaria induced by *Plasmodium falciparum*-infected mosquitoes. The Netherlands Journal of Medicine 63: 52-58.
14. Beier JC, Davis JR, Vaughan JA, Noden BH, Beier MS (1991) Quantification of *Plasmodium falciparum* sporozoites transmitted in vitro by experimentally infected *Anopheles gambiae* and *Anopheles stephensi*. The American Journal of Tropical Medicine and Hygiene 44: 564-570.
15. Gu W, Mbogo CN, Githure JI, Regens JL, Killeen GF, et al. (2003) Low recovery rates stabilize malaria endemicity in areas of low transmission in coastal Kenya. Acta Tropica 86: 71-81.
16. Reithinger R, Coleman PG, Alexander B, Vieira EP, Assis G, et al. (2004) Are insecticide-impregnated dog collars a feasible alternative to dog culling as a strategy for controlling canine visceral leishmaniasis in Brazil? International Journal for Parasitology 34: 55-62.
17. Mahmood F, Reisen WK (1981) Duration of the gonotrophic cycles of *Anopheles culicifacies* Giles and *An. stephensi* Liston, with observations on reproductive activity and survivorship during winter. Mosquito News 41: 22 - 30.
18. Krafsur ES (1977) The bionomics and relative prevalence of *Anopheles* species with respect to the transmission of *Plasmodium* to man in western Ethiopia. Journal of Medical Entomology 14: 180-194.
19. Gillies MT, Wilkes TJ (1965) A study of the age-composition of populations of *Anopheles gambiae* Giles and *A. funestus* Giles in North-Eastern Tanzania. Bulletin of Entomological Research 56: 237-262.
20. Gillies MT, Wilkes TJ (1963) Observations on nulliparous and parous rates in a population of *Anopheles funestus* in East Africa. Annals of Tropical Medicine and Parasitology 57: 204-213.
21. Krafsur ES, Armstrong JC (1982) Epidemiology of *Plasmodium malariae* infection in Gambella, Ethiopia. Parassitologia 24: 105-120.
22. Davidson G (1954) Estimation of the survival rate of anopheline mosquitoes in nature. Nature 174: 792-793.
23. Garrett-Jones C, Grab B (1964) The Assessment of Insecticidal Impact on the Malaria Mosquito's Vectorial capacity, from Data on the Proportion of Parous Females. Bulletin of the World Health Organization 31: 71-86.

24. Dye C (1992) The analysis of parasite transmission by bloodsucking insects. Annual Review of Entomology 37: 1-19.
25. Taye A, Hadis M, Adugna N, Tilahun D, Wirtz RA (2006) Biting behavior and *Plasmodium* infection rates of *Anopheles arabiensis* from Sille, Ethiopia. Acta Tropica 97: 50-54.
26. Deltinova TS (1962) Age-grouping methods in Diptera of medical importance. (WHO: Monograph Series, No. 47). Geneva: World Health Organization. 216 p.
27. Buykx CH (2000) Further Development of the "Insecticide Sponging of Livestock" Technique of Malaria Control. MSc Dissertation, London School of Hygiene and Tropical Medicine: University of London, UK.
28. Hewitt S, Kamal M, Muhammad N, Rowland M (1994) An entomological investigation of the likely impact of cattle ownership on malaria in an Afghan refugee camp in the North West Frontier Province of Pakistan. Medical and Veterinary Entomology 8: 160-164.
29. Sota T, Mogi M (1989) Effectiveness of zooprophylaxis in malaria control: a theoretical inquiry, with a model for mosquito populations with two bloodmeal hosts. Medical and Veterinary Entomology 3: 337-345.
30. Hadis M, Lulu M, Makonnen Y, Asfaw T (1997) Host choice by indoor-resting *Anopheles arabiensis* in Ethiopia. Transactions of the Royal Society of Tropical Medicine and Hygiene 91: 376-378.
31. Hewitt S, Rowland M (1999) Control of zoophilic malaria vectors by applying pyrethroid insecticides to cattle. Tropical Medicine & International Health 4: 481-486.
