# Supplementary material for: Controlling Malaria Using Livestock-Based Interventions: A One Health Approach
Source: PLoS One. 2014 Jul 22;9(7):e101699. doi: 10.1371/journal.pone.0101699 (PMC4106824; doi:10.1371/journal.pone.0101699)
Supplement: Text S4 — Sensitivity analyses. S4.1. Sensitivity analysis of the effects of untreated livestock. Figure S1. Sensitivity analysis of the effects of livestock availability/density vs. vector search-related mortality on several malaria outcomes, at the new endemic equilibrium. S4.2. Sensitivity analysis of the effects of insecticide-treated livestock. Figure S2. Sensitivity analysis of the effects of repellency/attractancy vs. insecticidal probability (k) on the prevalence ratio. S4.2.1. Sensitivity analysis of the impact of vector search-related mortality upon the effects of insecticide-treated livestock. Figure S3. Sensitivity analysis of the effects of repellency/attractancy vs. vector search-mortality on the prevalence ratio. Figure S4. Sensitivity analysis of the effects of repellency/attractancy vs. vector search-mortality on the critical proportion of insecticide-treated livestock. (PDF) [file pone.0101699.s004.pdf]

Text S4. Sensitivity analyses

Text S4.1. Sensitivity analysis of the effects of untreated livestock

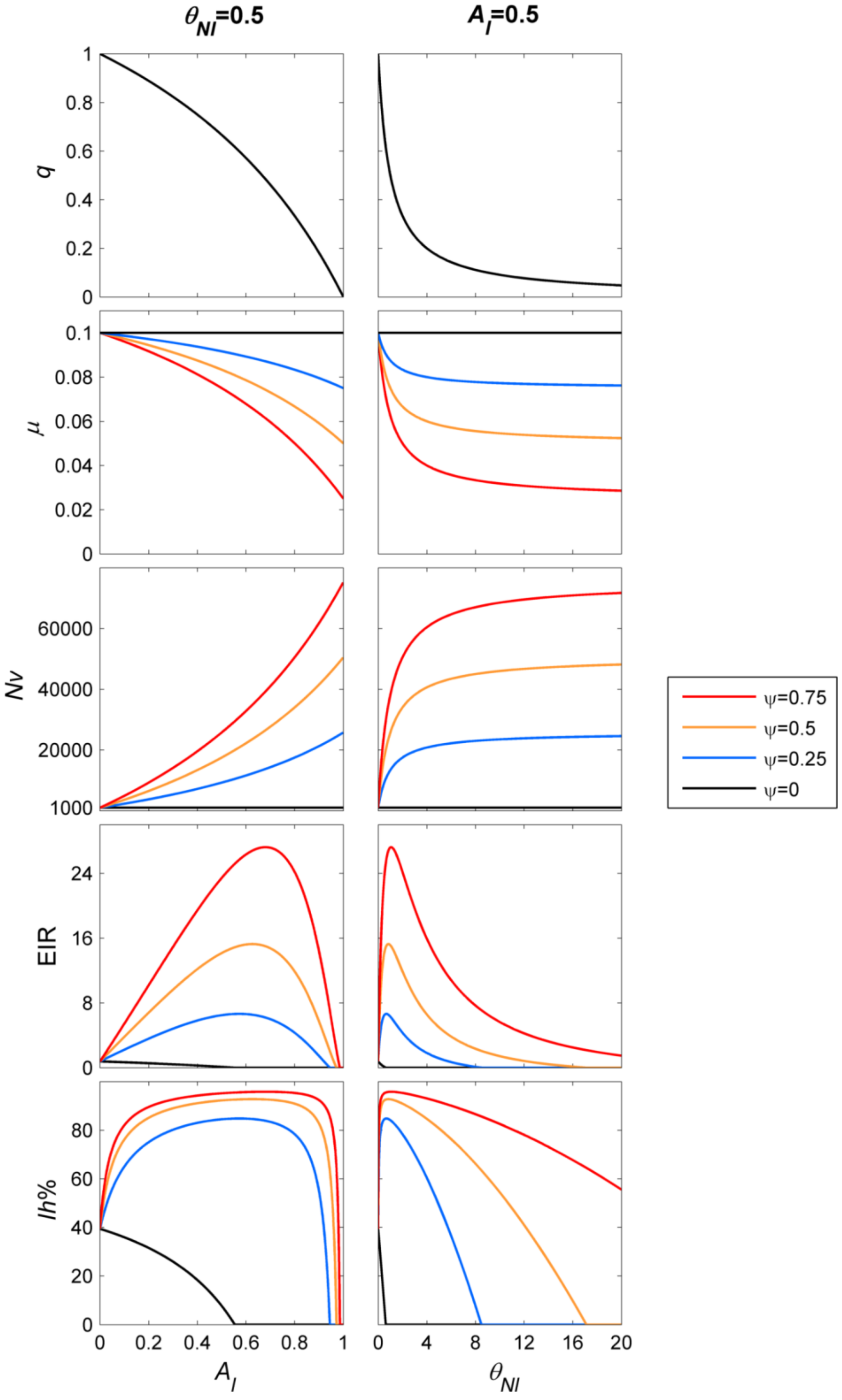

Figure S1. Sensitivity analysis of the effects of livestock availability/density vs. vector search-related mortality on several malaria outcomes, at the new endemic equilibrium

From black to red, there is increasing vector search-related mortality (modeled by increasing the proportion of the vector mortality pre-livestock introduction that is related

with host search (ψ)). The black line corresponds to the scenario where the search-related mortality pre-livestock introduction was negligible (ψ=0), and therefore introducing livestock has no impact on vector mortality or density. The orange line is equivalent to what is used in Figure 2 and Figure 3, where vector search-related mortality is half of the overall vector mortality pre-livestock introduction (ψ=0.5, baseline simulations). The effects of introducing livestock on the human blood index (q) are independent from the proportion of vector natural mortality that is related with host search. Additional outcomes: overall vector mortality rate (μ), vector density (N_v), entomological inoculation rate (EIR), and prevalence of human infection (I_h%). Left: 1 animal per every 2 persons (θ_{Nl}=0.5); along the x-axis is represented A_l, livestock availability to vectors. Right: the availability of livestock to vectors is the same as that of humans (A_l=0.5); along the x-axis, representing $\theta_{Nl}=N_l/N_h$, the livestock density N_l is varied relative to a fixed human density. N_h=100; $N_v(0)$ =1000; K=100,000. Other parameters are as in baseline simulations (Table 1).

Text S4.2. Sensitivity analysis of the effects of insecticide-treated livestock

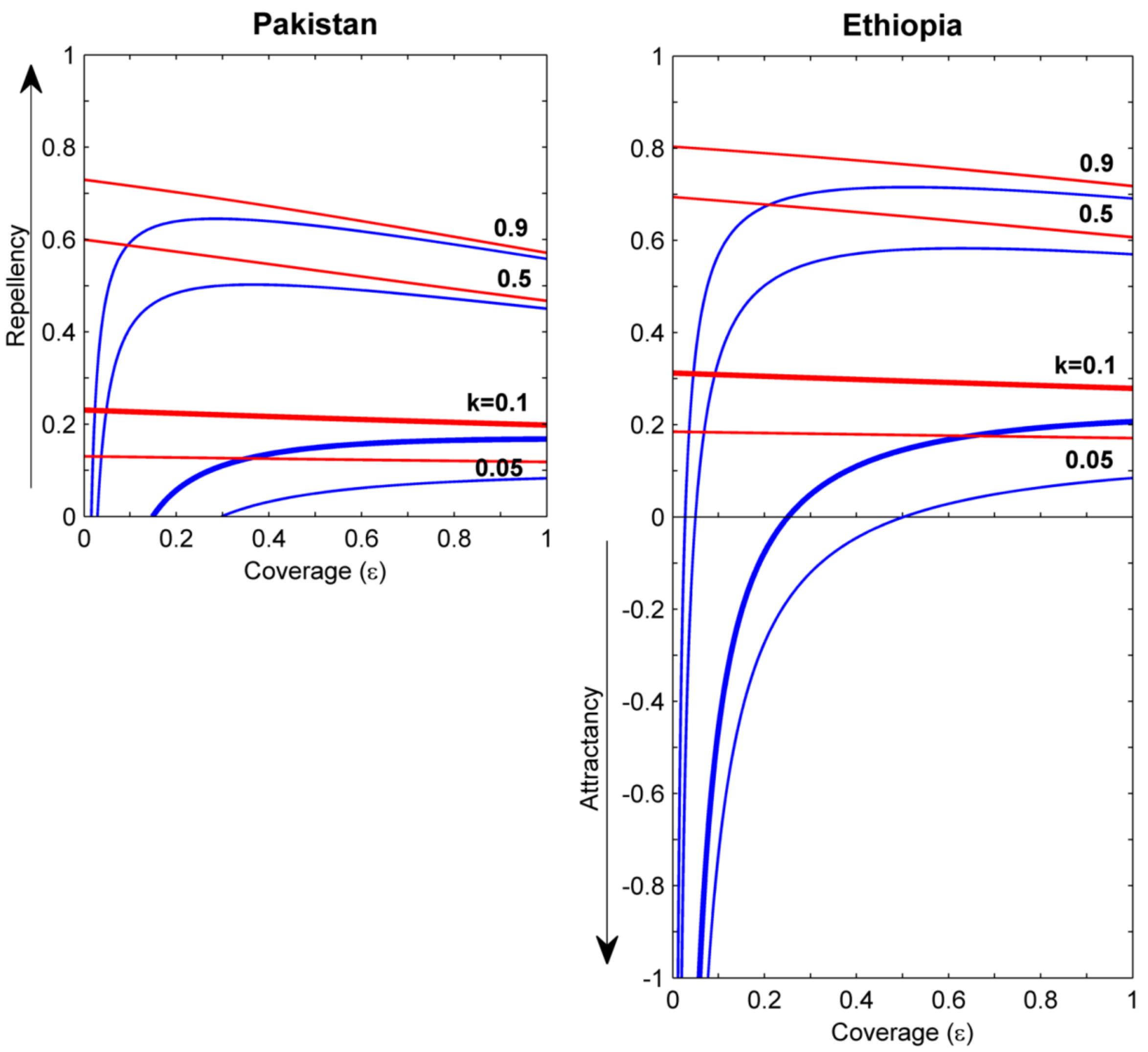

Figure S2. Sensitivity analysis of the effects of repellency/attractancy *vs*. insecticidal probability (*k*) on the prevalence ratio

This figure shows how the insecticidal probability (*k*) influences the effect of the insecticide diversionary properties on the proportion of ITL required to achieve a given prevalence ratio (prevalence with ITL / baseline prevalence). Blue lines: Prevalence ratio=0.46 (like the observed in the Pakistan trial); Red lines: Prevalence ratio=1 (above which treating livestock increases malaria prevalence). From bottom to top, *k* is increasing from 0.05, 0.1 (bold, baseline simulations), 0.5, up to 0.9. Along the y-axis the diversion probability, α, is varied (α >0, increasing repellency; α <0, increasing attractancy). Other parameters values as in baseline simulations (Table 2), namely, ψ'=0.5.

S4.2.1. Sensitivity analysis of the impact of vector search-related mortality upon the effects of insecticide-treated livestock

Despite there being uncertainty about the true value of the vector-search mortality, its magnitude only affects the impact of an ITL intervention if the insecticide has diversionary properties. The effects of varying the proportion of the vector natural mortality related with host search, ψ', can be understood better by looking closer at the expression for vector mortality rate when livestock are treated with insecticide:

$$\mu = \mu_m + \overbrace{\frac{a}{\left(N_h A_h + (1-\varepsilon\alpha)N_l A_l\right)j}}^{\mu_s} + \underbrace{\frac{\varepsilon(1-\alpha)N_l A_l}{N_h A_h + N_l A_l}ka}_{\mu_k}$$

and the equivalent expression when no livestock are treated:

$$\underbrace{\mu = \mu_m + \overbrace{\frac{a}{\left(N_h A_h + N_l A_l\right)j}}^{\mu_s}}_{\mu_0}$$

together with the considerations given in **Text S3.2** where letting $\mu_m = (1-\psi')\mu_0$ the expression for j becomes equivalent to:

$$j = \frac{a}{\left(N_h A_h + N_l A_l\right)\left(\mu_0 \psi'\right)}.$$

The lower the ψ' value, the higher is the vector minimum mortality rate (μ_m) and the higher is the value of j required to obtain a given observed μ_0. Higher j values result in smaller search-associated vector mortality. Thereby, decreases in the ψ' value will counteract the only benefit of repellence (which was an increase on the search-associated vector mortality), and consequently decrease the beneficial impact of an ITL intervention.

Overall, for a given repellency probability (α >0), the lower the ψ' value, the greater is the coverage required to achieve a given reduction in prevalence or in R_0, and the lower will be the repellency threshold above which ITL becomes deleterious (**Figure S3** and **Figure S4).**

For example, when k=0.1, if ψ'=0.5 (baseline simulations, bold line in **Figure S3**) the repellence threshold above which the intervention would become deleterious (α_c) is 0.20 for Pakistan and 0.28 for Ethiopia, while if ψ'=0.25 (lower background search-related mortality) it decreases to α_c=0.16 and 0.23, if ψ'=0 (null background search-related mortality) it further decreases to α_c=0.13 and 0.19 , and if ψ'=0.75 (higher background search-related mortality) it increases to α_c=0.27 and 0.36, for Pakistan and Ethiopia, respectively (**Figure S3**). When repellency is above 60% in Pakistan or Ethiopia, the

model predicts that the number of cases post intervention would always be higher than before, independently of the value of ψ' or coverage (**Figure S3**). Note that repellency has less detrimental impact on prevalence or on R_0 in Ethiopia than in Pakistan (**Figure 5**, **Figure S3** and **Figure S4**), which can be explained by the fact that repellence has also less impact on μ_s in Ethiopia than in Pakistan [1].

Conversely, if the insecticide has attractancy properties (α <0), the lower the ψ' value, the smaller is the coverage required to achieve a given reduction in prevalence or R_0 (**Figure S3** and **Figure S4).**

In general, the smaller the background search-related vector mortality (pre-livestock treatment), the greater is the critical coverage if there is repellency (for any given α >0), and the lower is the critical coverage under attractancy (for any given α <0) (**Figure S4)**. This relationship becomes however increasingly non-linear with increase in the insecticidal properties (k) of a treatment with repellency applied to livestock, with more marked non-linearity in Pakistan. For example, when k=0.5 in the Asian setting, if ψ'=0.3 and α =0.4, when the coverage is >30% and <85 % then R_0<1, but for higher coverage then R_0 becomes >1 (**Figure S4E)**.

References

1. Franco AIO (2010) Effects of livestock management and insecticide treatment on the transmission and control of human malaria. PhD thesis. London School of Hygiene and Tropical Medicine, University of London, United Kingdom.

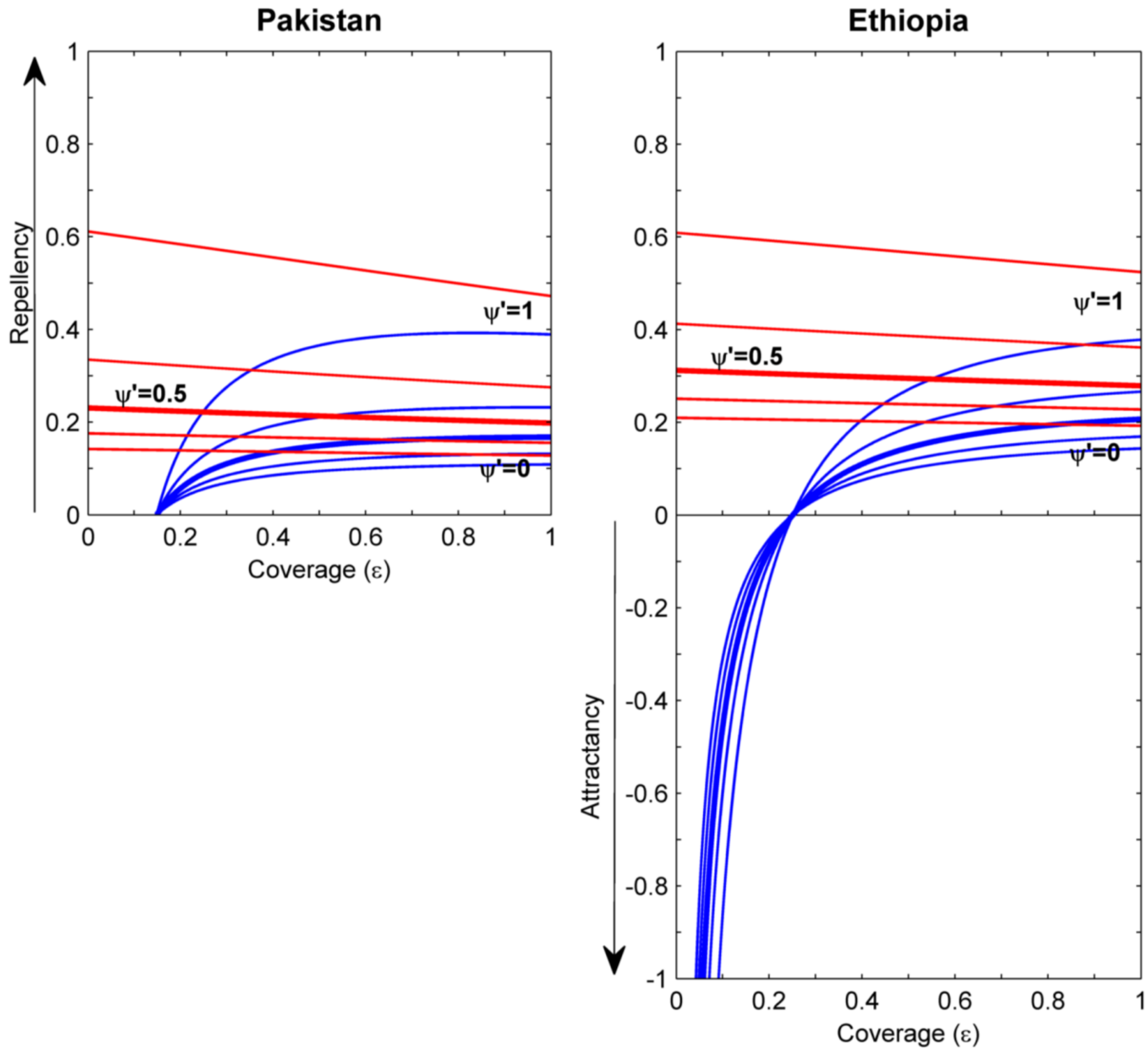

Figure S3. Sensitivity analysis of the effects of repellency/attractancy *vs.* vector search-mortality on the prevalence ratio

This figure shows how the vector search-mortality influences the effect of the insecticide diversionary properties on the proportion of ITL required to achieve a given prevalence ratio (prevalence with ITL / baseline prevalence). Blue lines: Prevalence ratio=0.46 (like the observed in the Pakistan trial); Red lines: Prevalence ratio=1 (above which treating livestock increases malaria prevalence). From bottom to top, the proportion of the natural vector mortality related with host-search (ψ') is increasing from 0, 0.25, 0.5 (bold, baseline), 0.75, up to 1. Along the y-axis the diversion probability, α, is varied (α >0, increasing repellency; α <0, increasing attractancy). Other parameters values as in baseline simulations (Table 2), namely, k=0.1.

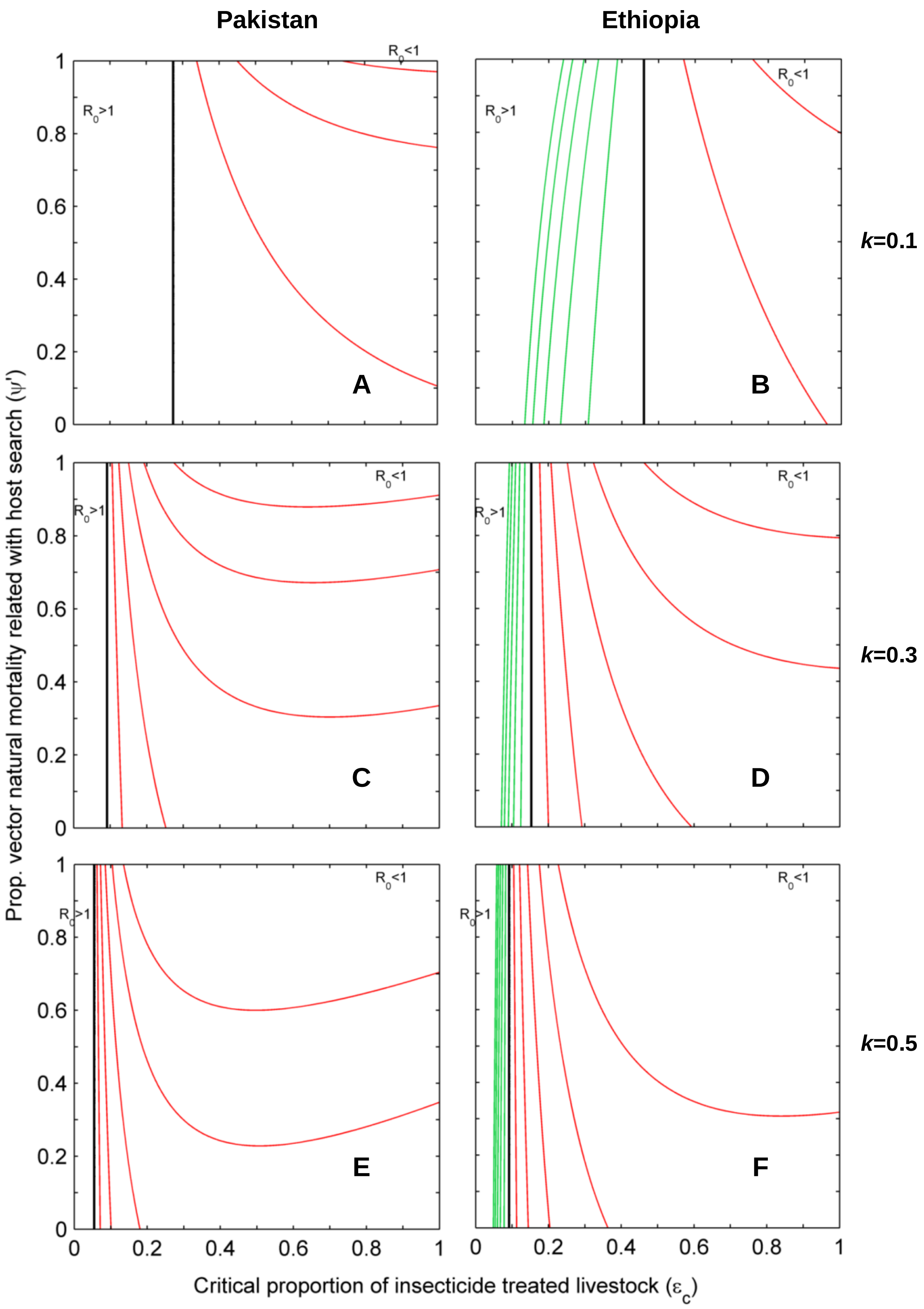

Figure S4. Sensitivity analysis of the effects of repellency/attractancy *vs.* vector search-mortality on the critical proportion of ITL

The lines show the combination of values of coverage and proportion of vector natural mortality related with host search (ψ') required to achieve R_0=1, above which R_0 will be decreased below 1, for a given diversion probability (α). Black Line: insecticide without repellence or attractancy (α=0, baseline). Red lines: repellency increasing towards the right (α increasing from 0.1, to 0.2 , 0.3, 0.4, 0.5) . Green lines: attractancy increasing towards the left (α decreasing from -0.1, to -0.2, -0.3, -0.4, -0.5). Other parameter values as in baseline simulations (Table 2).
